# Manipulating Interfacial Stability via Preferential Absorption for Highly Stable and Safe 4.6 V LiCoO_2_ Cathode

**DOI:** 10.1007/s40820-025-01694-4

**Published:** 2025-03-12

**Authors:** Long Chen, Xin He, Yiqing Chen, Youmin Hou, Yujie Zhang, Kangli Wang, Xinping Ai, Yuliang Cao, Zhongxue Chen

**Affiliations:** 1 Key Laboratory of Hydraulic Machinery Transients, Ministry of Education, School of Power and Mechanical Engineering, Wuhan University, Wuhan, 430072 People’s Republic of China; 2 State Key Laboratory of Advanced Electromagnetic Technology, School of Electrical and Electronic Engineering, Huazhong University of Science and Technology, Wuhan, 430074 People’s Republic of China; 3 Hubei Key Laboratory of Electrochemical Power Sources, College of Chemistry and Molecular Sciences, Wuhan University, Wuhan, 430072 People’s Republic of China

**Keywords:** Electrolyte design, LiF-rich interface, Wide-temperature, High-safe, 4.6 V LCO

## Abstract

Elevating the upper cutoff voltage to 4.6 V could effectively increase the reversible capacity of LiCoO_2_ (LCO) cathode, whereas the irreversible structural transition, unstable electrode/electrolyte interface and potentially induced safety hazards severely hinder its industrial application. Building a robust cathode/electrolyte interface film by electrolyte engineering is one of the efficient approaches to boost the performance of high-voltage LCO (HV-LCO); however, the elusive interfacial chemistry poses substantial challenges to the rational design of highly compatible electrolytes. Herein, we propose a novel electrolyte design strategy and screen proper solvents based on two factors: highest occupied molecular orbital energy level and LCO absorption energy. Tris (2, 2, 2-trifluoroethyl) phosphate is determined as the optimal solvent, whose low defluorination energy barrier significantly promotes the construction of LiF-rich cathode/electrolyte interface layer on the surface of LCO, thereby eventually suppresses the phase transition and enhances Li^+^ diffusion kinetics. The rationally designed electrolyte endows graphite||HV-LCO pouch cells with long cycle life (85.3% capacity retention after 700 cycles), wide-temperature adaptability (− 60–80 °C) and high safety (pass nail penetration). This work provides new insights into the electrolyte screening and rational design to constructing stable interface for high-energy lithium-ion batteries.

**Supplementary Information:**

The online version contains supplementary material available at 10.1007/s40820-025-01694-4.

## Highlights

- A novel electrolyte design strategy for high voltage and high safe LiCoO_2_ (LCO) cathode based on highest occupied molecular orbital and LCO absorption energy descriptor was proposed.
- The irreversible phase transformation was restricted by the LiF rich LCO/electrolyte interface.
- The well designed tris 2, 2, 2-trifluoroethyl phosphate electrolyte endows Ah grade Gr||LCO pouch cell with excellent electrochemical performance (85.3% capacity retention after 700 cycles), low-temperature adaptability (−60 °C retention: 53%) and greatly improved thermal safety (pass nail penetration).

**Supplementary Information:**

The online version contains supplementary material available at 10.1007/s40820-025-01694-4.

## Graphical abstract

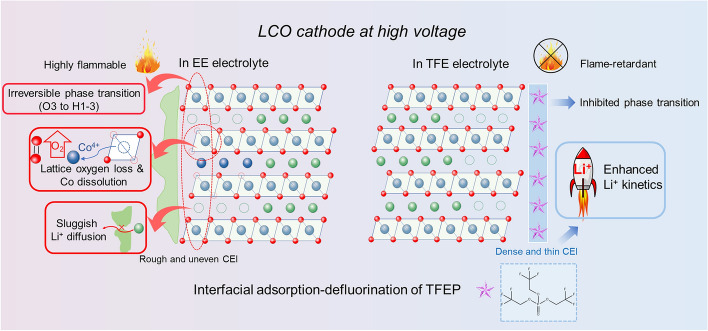

## Introduction

**Fig. 1:**
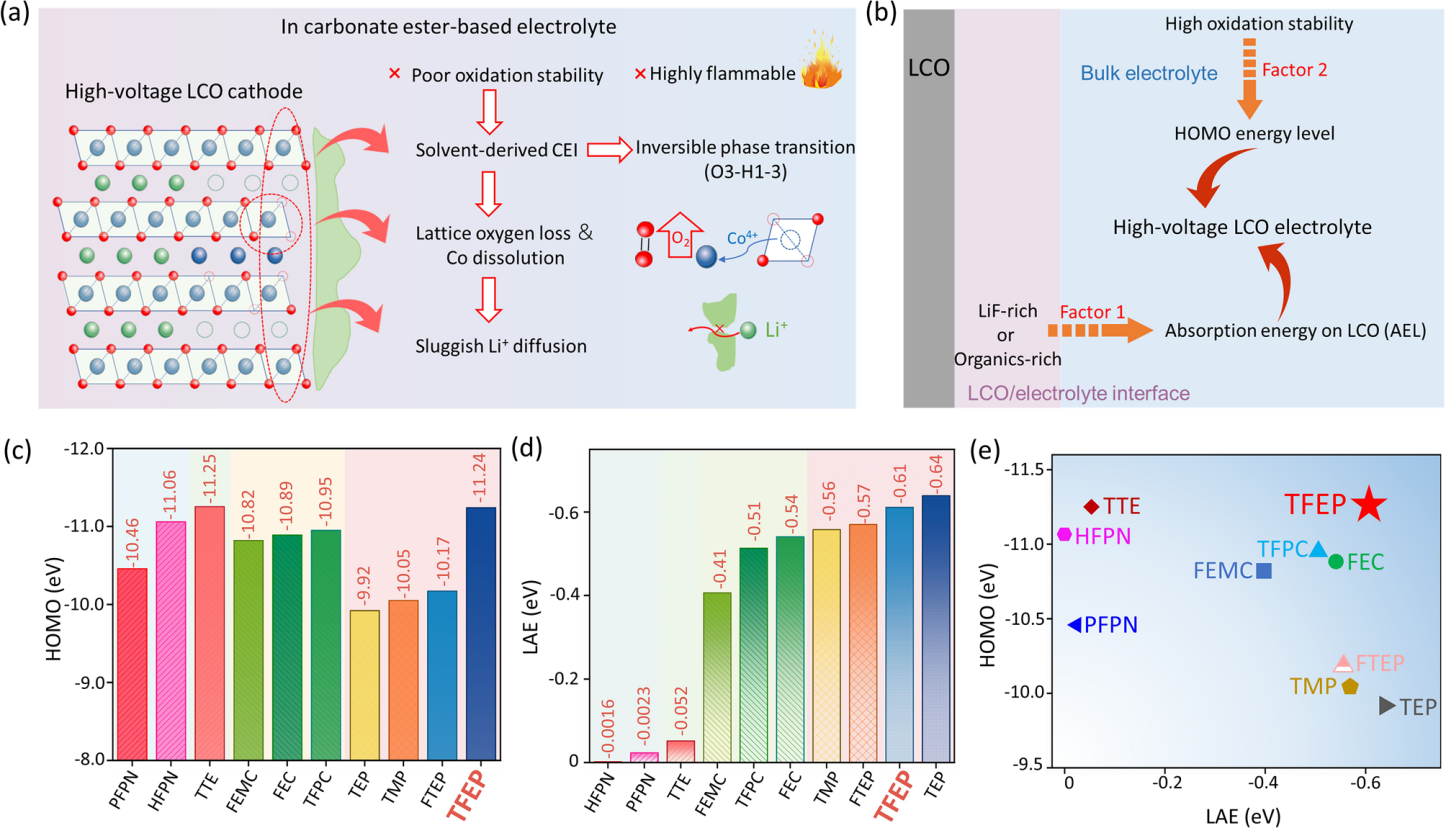
Dilemma of HV-LCO and electrolyte design principle. **a** Dilemma of HV-LCO with EC-based electrolytes; **b** solvent selection principle; **c** HOMO energy levels of ten typical flame-retardant solvents; **d** LAE of different solvents; **e** two-dimensional figure for solvent selection, while *X*-axis is LAE and *Y*-axis is HOMO energy level

Lithium cobalt oxide (LiCoO_2_), as one of the most popular cathode materials, has overwhelmingly dominated the lithium-ion battery (LIB) markets of consumer electronics due to its high theoretical capacity, favorable operating voltage and volumetric energy density [1–4]. To cope with the energy density demands of the ever-expanding information and communication revolutions, researchers have attempted to elevate the energy density of LIBs by charging the LiCoO_2_ to a higher voltage above 4.5 V [5, 6]. However, increasing the charging cutoff voltage induces the detrimental phase transition of LiCoO_2_ from O3 to H1-3 phase [7, 8]. Worse still, traditional carbonate solvents (such as ethylene carbonate) suffer from oxidation and dehydrogenation at higher voltage triggered by the catalytic action of high oxidation state of Co^4+^ ions, forming an instable cathode/electrolyte interface (CEI) [9–11]. The dehydrogenation of the solvent generates highly corrosive HF, which accelerates the dissolution of Co and the collapse of LiCoO_2_ structure, and eventually leads to severe capacity decay [12, 13] (Fig. 1a). The above interface instability issue is more prominent when LiCoO_2_-based batteries are operated or stored at high temperature, which will bring more serious safety hazards.

Extensive efforts have been devoted to easing these issues of high-voltage LiCoO_2_ (HV-LCO). The most explored methods are surface coating [14–17] and elemental doping [18–23], which could improve the stability of Co–O bond, prevent the surface degradation and inhibit the irreversible phase transition, but the structural modification (especially coating) also increases the complexity of industrial production [17] and is unable to affect the intrinsic safety of the battery system. Compared to the modification of LCO electrode, electrolyte engineering is a more straightforward and efficient approach to improve the LCO/electrolyte interface property. Highly concentrated electrolytes and all-fluorinated solvents aid the formation of LiF-rich high oxidation stability CEI interfaces [24–26], but the high viscosity and high cost would heavily restrain their practical applications, especially at low temperature. Use of electrolyte additives such as sulfur, silicon and phosphorus-containing species is a traditional but functional strategy to improve the stability of HV-LCO. However, similar to structural modification, additives also show little impact on the enhancement of safety capacity [27–30]. It has been well recognized that the CEI film-forming reaction on the HV-LCO surface is more intricate than the simple solvent reduction film-forming behavior on the surface of the graphite anode [31–34], because it is affected by the catalytic action of the high valence Co and high chemical reactivity of oxygen species. This elusive interfacial chemistry poses substantial challenges to the rational design of highly compatible electrolytes for HV-LCO cathode.

In this work, we propose a novel electrolyte design strategy to realize long-life, high-safe and wide-temperature-range HV-LCO cathode: first, establishing a flame-retardant solvent library (including phosphazenes, fluorinated carbonate esters/ethers and phosphate esters), whereafter assessing these solvents by a dual descriptor: highest occupied molecular orbital (HOMO) energy levels (corresponding to the oxidation limit of solvents) and LCO absorption energy (LAE, the preferential absorption on LCO surface could dominate the CEI property). Tris (2, 2, 2-trifluoroethyl) phosphate (TFEP) was selected as the optimal solvent; its low defluorination energy barrier (far smaller than dehydrogenation) could promote the construction of F-rich passivation layer on the surface of LCO. The robust LCO/electrolyte interface prominently consolidates the surface lattice and bulk structure of LCO, resists the irreversible phase transition, suppresses adverse side reactions and facilitates the Li^+^ transportation. Besides, the nonflammable TFEP-based electrolyte (denoted as TFE) can greatly enhance the battery safety. As a result, the graphite||LCO pouch cell achieves an excellent cycling stability (85.3% capacity retention after 700 cycles), wide-temperature adaptability (− 60–80 °C) and high safety (pass the nail penetration test). Although TFEP has been reported before, the significant difference from previous works is that this work identified the superiority of TFEP in HOMO energy level and LAE compared with many other commonly used solvents for the first time, which is beneficial to the construction a robust LiF-rich CEI layer on the surface of LCO. With regard to the profits from the robust CEI layer, this work provides new insights into manipulating the electrolyte chemistry to constructing stable interface for rechargeable batteries.

## Experimental Section

### Materials

The electrode materials including LiCoO_2_ and graphite (Gr), and high purity solvents FEC, EMC, and lithium salts LiPF_6_ were purchased from Canrd Technology Co. Ltd. All of them were used directly without further purification. The TFEP solvent was purchased from Shanghai Bide Pharma Tech Co., Ltd. and was purified with a redistillation step under vacuum after dehydration with CaH_2_ under an atmosphere of inert gas for 12 h. All the electrolytes in the paper were prepared in the MIKROUNA glovebox filled with Ar gas. The anode was composed of 85% Gr, 6% Super P, 4.5% sodium carboxymethyl cellulose (CMC) and 4.5% styrene butadiene rubber (SBR). The cathode was composed of 90% LCO, 5% Super P and 5% polyvinylidene fluoride (PVDF), and the areal loading of cathode materials is ~ 11 mg cm^−2^ (the total mass loading of the electrode is 12–13 mg cm^−2^).

### Electrochemical Measurements

All the coin cells mentioned in the paper were assembled by CR2032 coin cells in the argon-filled glovebox. The coin cells were tested on battery testers (CT2001A, Land Wuhan, China). Linear sweep voltammetry (LSV) and electrochemical impedance spectroscopy (EIS) were carried out with an electrochemical workstation (CS350H, Corrtest Wuhan, China). The 0.7 and 3.6 Ah Gr||LCO pouch cell was assembled by EVE Energy Co., Ltd. The mass loadings of the Gr anode and the LCO cathode were 20.5 and 36.1 mg cm^−2^, respectively. The pouch cells were tested on a Neware battery test system (CT-4008–5V20A-A, Shenzhen, China). Safety tests on the pouch cells were carried out according to IEC 62660–4 requirements.

### Characterizations

The surface chemistry was acquired by X-ray photoelectron spectroscopy (XPS, Thermo Scientific ESCALAB250Xi). The surface morphology of electrode was acquired by scanning electron microscopy (SEM, ZEISS Merlin Compact VP), focus ion beam-scanning electron microscope (FIB-SEM, SOLARIS GMH) and spherical aberration corrected transmission electron microscope (AC-TEM, JEOL JEM-ARM300F). The ionic conductivity of electrolytes was measured by the conductivity meter (DDS-307, Leici, China) at different temperatures (from − 60 to 80 °C). The characterizations of structural and phase transformation of LCO were investigated by Operando X-ray diffraction (XRD, Rigaku Ultima IV, Japan, λ = 0.154056 nm) and Operando Raman spectrum (XploRA plus).

Operando XRD (Rigaku Ultima IV, Japan, λ = 0.154056 nm) patterns were measured to monitor the real-time phase transition of LCO during the cycling process, and the tests were performed in an in situ X-ray diffraction lithium-ion battery (LIB-XRD, purchased from Beijing Scistar Technology Co. Ltd). The scan rate was set as 1° min^−1^ in the degree range of 17–23° (2-theta).

The operando Raman measurements were taken by a Renishaw InVia Raman microscope with a 633-nm wavelength laser; CR2032-type coin cell with a small hole covered by a 2-mm-thick sapphire was assembled to ensure the irradiation of the LCO surface through Li metal foil and a glass fiber separator.

### Computational Methods

In this work, theory calculations were performed using the Vienna ab Initio Simulation Package (VASP) code. The LiCoO_2_ (014) surface with a thickness of 15 Å vacuum slab, which avoid layer-to-layer interaction for periodic structure, was built for subsequent calculations. Some pseudo-H atoms were attached to the lowermost layer of the LCO model to saturate the dangling bonds. Subsequent calculations were done on the uppermost surface. For geometry optimization, the cutoff energy for plane wave basis set was set to 500 eV. For slow-growth molecular dynamics (SGMD), the cutoff energy for plane wave basis set was set to 400 eV. All calculations in this work, Gaussian smearing, were used. The smearing value, self-consistent field energy and ionic force convergence tolerance were set to 0.1 eV, 1 × 10^–6^ eV and 0.02 eV Å^−1^, respectively. The electron core interactions were treated with the projector-augmented wave (PAW) pseudopotential, and Perdew–Burke–Ernzerhof (PBE) of the generalized gradient approximation (GGA) scheme was carried out for exchange–correlation potential. For Brillouin zone sampling, Γ point was used.

In order to compare the dissociation free energy change of molecules on the LCO surface, the thermodynamic integration method was performed to calculate the free energy change. Specifically, slow-growth molecular dynamics (SGMD) calculations were performed on LiCoO_2_ (014) surface with adsorbed TFEP. For the dissociation of H atoms, the collective variable (CV) was defined as the distance between H atom and connected C atom. And for the dissociation of F atoms, the collective variable (CV) was defined as the distance between F and the surface Co atom minus the distance between C atoms attached to the F atom. The step size of MD is 1 fs, and the control temperature is 298 K. The CV increases by 0.0005 angstroms in each step, which corresponds to the dissociation of H or F atom from the molecule. The slow-growth approach has been implemented by VASP code and performed a home-madecodes/script to post-process the VASP data.Quantum chemical calculations were carried out with ORCA (version 5.0.3). Molecular structures were optimized using the B97-3c functional with the def2-TZVP basis set. Single-point energy calculations were performed with the M06-2X functional, also using the def2-TZVP basis set, and included the D3ZERO dispersion correction. Results from these calculations were further analyzed using Multiwfn for post-processing.

## Results and Discussion

### Solvent Assessment

The electrolytes that can endow HV-LCO with favorable cycling stability and high safety are fain to follow requirements below: (i) possessing a high oxidation potential, thereby inhibiting the malignant decomposition of the electrolyte at high voltage, (ii) forming a dense and robust CEI film on the LCO surface, which could isolate LCO from electrolytes, eventually suppress the irreversible phase transition and boost enhanced Li^+^ transport kinetics, (iii) owning flame-retardant properties.

Figure S1 displays the planar-averaged total potential energy of LCO with ethylene carbonate (EC) absorbed on the surface along the Z direction; clearly, LCO exhibits a deeper potential compared to EC at the LCO/electrolyte interface. This disparity facilitates the transfer of electrons from LCO to solvents, as electron transfer spontaneously occurs from regions of lower potential to higher potential. Hence, the solvent with high LCO absorption energy (LAE) will determine the property of LCO/electrolyte interface. Besides, the solvents are anticipated to own low HOMO energy level, so that they can render the electrolyte higher oxidation resistance (Fig. 1b). Hence, a comprehensive assessment of ten typical flame-retardant solvents (the full name and physical properties are shown in Table S1) based on their HOMO and LAE was performed by density functional theory (DFT) calculations.

Figures 1c and S2 depict the HOMO energy levels of the solvents and their corresponding chemical structures. In general, fluorinated solvents exhibit lower HOMO energy levels due to the strong electron-withdrawing effect of the fluorine element. Among these ten solvents, 1, 1, 2, 2-tetrafluoroethyl 2, 2, 3, 3-tetrafluoropropylether (TTE) and TFEP have the lowest HOMO values of − 11.25 and − 11.24 eV, indicative of the splendid capability to improve the oxidation resistance of the electrolyte system.

Turning to the value of LAE (Figs. 1d and S3), ester solvents with symmetrical molecular structures show stronger tendency to absorb on LCO surface. Despite the large steric hindrance caused by fluorine substitution, TFEP still exhibits comparable LAE metrics (− 0.61 eV) to TEP (− 0.64 eV), significantly outperforming other solvents. Based on the two-dimensional combinatorial screening of HOMO and LAE, TFEP was thereby determined as the optimized solvent for further investigation (Fig. 1e).

### Formation of LiF-Rich Electrode/Electrolyte Interface

Considering the low viscosity (0.65 cP at 25 °C) and low melting point (− 55 °C) that are desirable for wide-temperature-range applications, ethyl methyl carbonate (EMC) was selected as the co-solvent for TFEP. Hence, 1 mol L^−1^ LiPF_6_-TFEP/EMC (3:7 by vol., denoted as TFE) was designed as the optimal electrolyte, and 1 mol L^−1^ LiPF_6_-EC/EMC (3:7 by vol.) was chosen as base electrolyte (named as EE).

**Fig. 2:**
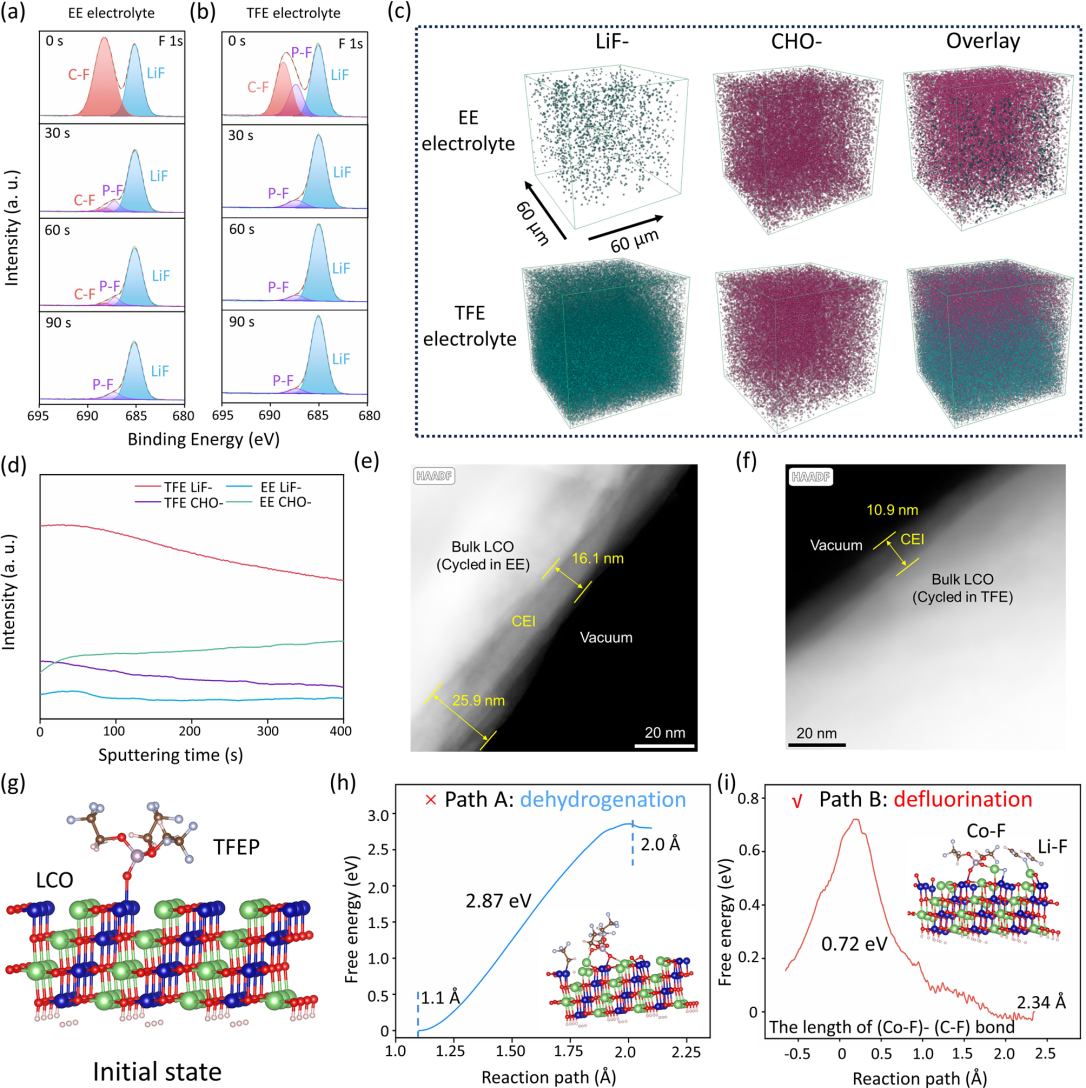
Characteristics and mechanism of TFEP-induced electrode/electrolyte interface. **a-b** F 1*s* spectra of CEI film formed on LCO surface in **a** EE and **b** TFE electrolyte with different etching time; **c** 3D distribution of LiF-, CHO- and overlay resolved by TOF–SIMS in EE and TFE electrolytes; **d** depth files of LiF- and CHO- in different electrolytes; HAADF-STEM pictures of CEI films formed in **e** EE and **f** TFE electrolytes; **g** initial absorption model of TFEP on LCO; **h** dehydrogenation process; **i** defluorination process

Forasmuch as the cycling performance of HV-LCO is heavily dominated by the electrode/electrolyte interface, series of characterizations were conducted to deeply investigate the discrepancies between CEI films formed in EE and TFE electrolytes. The chemical composition of CEI was first inspected by XPS etching technique. As seen in Fig. 2a, b, the relative content of inorganic components (LiF and P-F bond, attributed to the decomposition products of TFEP solvents and PF_6_^−^ anions) in TFE-derived CEI film is obviously higher than that in EE electrolyte-derived CEI layer. With the increase of etching time, a certain proportion of C–F bond still exists in the inner layer of CEI film formed in EE electrolyte, which would damage the mechanical stability and Li^+^ diffusion kinetics. Figure S4 reveals the atomic ratios (C, F, O, P) of CEI layers on LCO surface at different etching time. Obviously, the CEI derived from TFE contains more F element, especially with increased etching depth, roughly twice that of EE electrolyte, implying the formation of a F-rich CEI film on the surface of cycled LCO in TFE electrolyte.

Time-of-flight secondary ion mass spectrometry (TOF–SIMS) was further employed to analyze the compositions of CEI films. Considering that LiF has been commonly recognized as a favorable component of CEI film, we chose LiF- and CHO- (the characteristic ionic fragments of LiF and organic components) as the monitoring targets. Figure 2c exhibits the three-dimensional (3D) structure and spatial distribution of LiF- and CHO- fragments. Notably, the distribution of LiF- species was quite scarce and uneven in the CEI films formed in EE electrolyte, instead CHO- based organic components dominated the CEI film. In sharp contrast, a fairly uniform and dense distribution of LiF- species could be perceived along the lateral and thickness direction in TFE system, and the overlay image confirmed that the film generated in TFE electrolyte was composed of outer organic layer and inner inorganic layer. As well demonstrated, this CEI structure possesses merits of both mechanically stable and flexible, which is essential for the stable cycling of HV-LCO. The depth profiles of LiF- and CHO- fragments are displayed in Fig. 2d. The LiF- content in TFE system remains several times higher than that in EE electrolyte systems within 400 s sputtering depth, proving that a F-rich CEI film was constructed on the LCO surface induced by the introduction of TFEP.

The superiority of F-rich CEI film was ulteriorly investigated by high-angle annular dark field-scanning transmission electron microscopy (HAADF-STEM) and electrochemical impendence spectroscopy (EIS). As shown in Fig. 2e, an uneven CEI film with a thickness ranging from 16.1 to 25.9 nm was observed on the surface of LCO cycled in EE electrolyte. By contrast, an exceedingly homogeneous CEI film with a thickness of 10.9 nm was formed in TFE electrolyte (Fig. 2f). Additionally, the elemental distribution analysis of the CEI was further identified by STEM-EDS (Fig. S5) and two characteristic elements were selected for the observation: Co (represents LCO, in blue) and F (represents interface, in yellow). As clearly seen in Fig. S5a–c, F content in EE-derived CEI film is quite low and unevenly distributed, which is consistent with the TOF–SIMS and XPS results. Besides, due to the limited shielding ability of the organic-rich CEI to the electron beam, obvious Co element distribution is also observed near the interface (but the intensity was weakened compared with that of bulk LCO), which is attributed to the underlying LCO. On the contrary, the CEI formed in TFE electrolyte exhibits a fairly uniform distribution of F element with rich content (Fig. S5d, e), indicating that the preferential absorption of TFEP on LCO surface facilitates the formation of a thin and dense inorganic LiF-dominated CEI.

As for EIS measurements, Fig. S6b, c shows the Nyquist plots at different cycles, and the characteristic parameters fitted by the equivalent circuit (Fig. S6a) are listed in Table S2. As is seen, the initial charge-transfer impedance (*R*_ct_) in TFE was slightly larger than that in EE due to the larger viscosity and lower ionic conductivity of TFE. On the contrary, the *R*_ct_ in TFE (61.7 Ω) is significantly lower than that in EE (224.3 Ω) after one cycle, indicating the faster reaction kinetics at the interface. Besides, benefiting from the excellent ionic transportation property in LiF-rich CEI film, the impedance of CEI film (*R*_CEI_) formed in TFE electrolyte (49.8 Ω) is also far lower than that in EE electrolyte (203.5 Ω). After 50 cycles, the *R*_CEI_ and *R*_ct_ in TFE electrolyte almost keep unchanged, suggesting the exceptional stability of the electrode/electrolyte interface. In contrast, both *R*_CEI_ and *R*_ct_ in EE electrolyte increase sharply.

The mechanical stability of CEI films was assessed by Young’s modulus (calculated by AFM). As shown in Fig. S7, the largest and average Young’s modulus of CEI film (44.4/8.7 GPa) formed in TFE is nearly three times that of CEI in EE electrolyte (17.8/3.2 GPa), indicative of the enhanced mechanical stability. To more intuitively demonstrate the differences between EE and TFE-derived CEI film, the distribution of Young’s modulus is shown in Fig. S8. Besides, it has been widely recognized that the soft organic components can improve the flexibility of CEI. Therefore, the inorganic/organic hybrid construction in this work endows CEI with a combination of rigidity and flexibility.

In view of the difference in CEI between the two cells that is solely caused by the substitution of TFEP for EC, hence the role of TFEP in improving the interface stability was then deeply explored through theoretical calculations. Generally, there are primarily two decomposition pathways for TFEP solvents absorbed on the LCO surface: defluorination or dehydrogenation. The simulated model of TFEP-LCO compound is illustrated in Fig. 2g. In order to compare the difference in dissociation free energy of two pathways, slow-growth molecular dynamics (SGMD) calculations [35, 36] were performed on LCO surface (014) crystal plane with adsorbed TFEP. The final results are presented in Fig. 2h, i; the changes in the collective variable (CV) correspond to the breaking of the C-H bond and C-F bond in TFEP, respectively. By controlling the variation of the CV, the free energy changes during the bond-breaking process were recorded. As formulated in Fig. 2h, the free energy of dehydrogenation process is 2.87 eV, while the free energy of the whole defluorination process (involving the cleavage of the C–F bond and the formation of the Co–F bond) is only 0.72 eV, far lower than that of the dehydrogenation process, suggesting that the TFEP absorbed on the surface of LCO is prone to defluorinate rather than dehydrogenate. On the whole, the above experimental results and theoretical simulations fully verify that the introduction of TFEP hastens the formation of a robust F-rich CEI film and improves the stability of the interface.

### Inhibited Irreversible Phase Transition

**Fig. 3:**
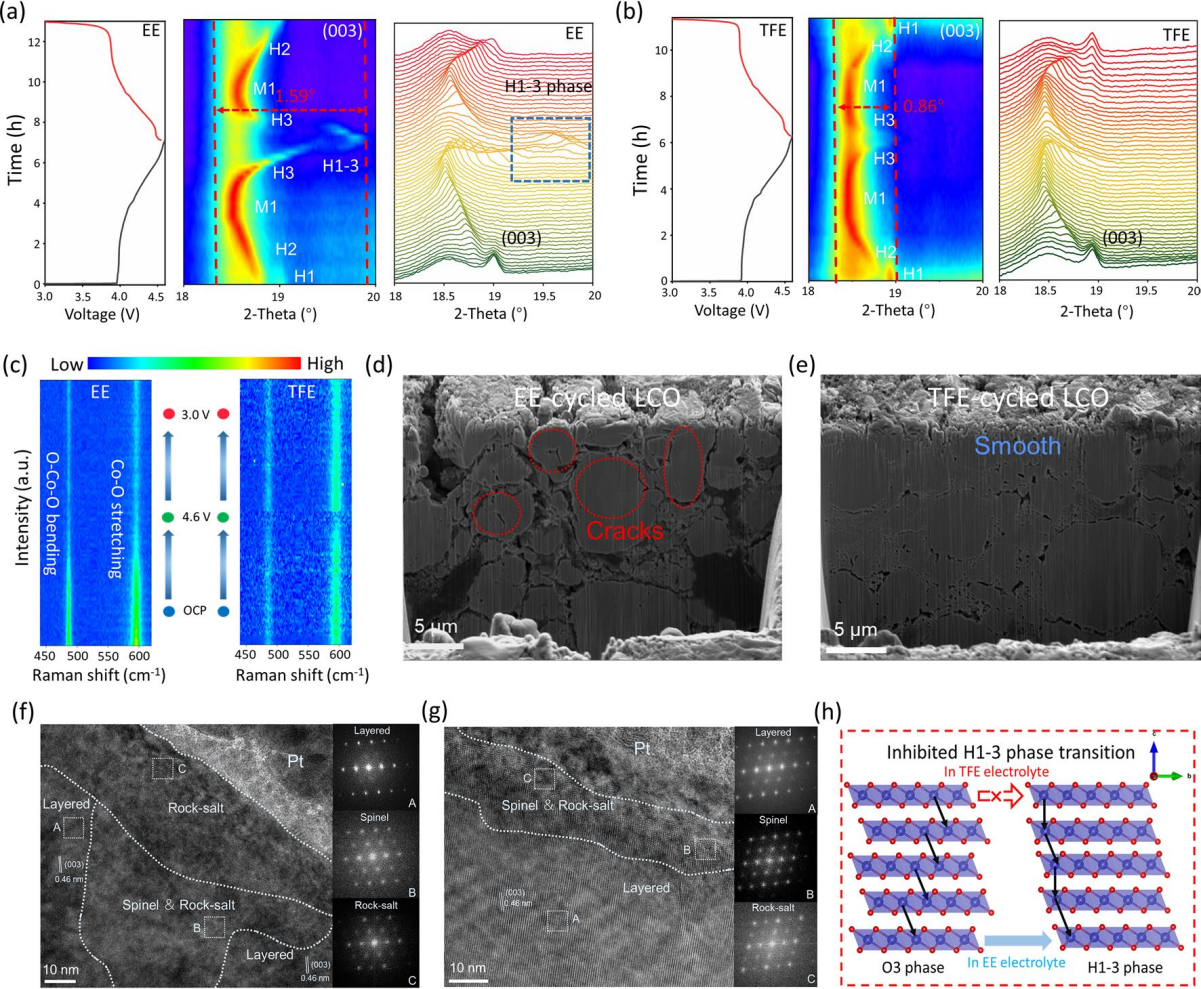
Characterizations of phase and structural transformation in different electrolytes. *In situ* XRD patterns of LCO in **a** EE and **b** TFE electrolytes in the first cycle; **c** *in situ* Raman spectra of LCO cathodes in EE and TFE electrolytes during initial cycle; FIB-SEM pictures of **d** EE-cycled and **e** TFE-cycled electrodes; **f** HR-TEM images of LCO particles after 20 cycles with a voltage range from 3.0 to 4.6 V in **f** EE and **g** TFE, the corresponding FFT patterns were shown inset; **h** simplified working mechanism of TFE electrolyte

The evolution from the electrochemically favorable O3 phase to the detrimental H1-3 phase under high operating voltage leads to the capacity attenuation of HV-LCO cathodes, while the stability of LCO superficial lattice plane depends largely on the CEI film formed on the LCO surface. Hence, the impact of CEI film on the structural transformation of LCO during the first cycle is further explored. Figure 3a, b shows the *in situ* XRD patterns and the contour maps. Herein the (003) peak located at 19.0° is chosen as the typical symbol to reflect the phase transition during cycling [7]. In EE electrolyte, the phase transition from O3 phase to H1-3 phase is clearly observed after charging to 4.6 V (Fig. 3a); howbeit, the transition is substantially inhibited in TFE system (Fig. 3b). The inhibited transition was further proved by the change of lattice spacing related to the (003) peak. As shown in Fig. S9, the initial lattice spacing of LCO is 0.466 nm. Upon charging, the (003) lattice spacing of EE-LCO shows an obvious decrease at high voltage (0.478 to 0.447 nm), indicating the phase transition from H3 to H1-3 phase. The decreased lattice spacing will weaken the Li^+^ kinetics in LCO, while in TFE-LCO, the malignant phase transition is effectively restrained (0.477 to 0.465 nm) benefiting from the LiF-rich CEI film. The attenuation ratio of lattice spacing reduces from 6.65% (EE-LCO) to 2.57% (TFE-LCO).

*In situ* Raman spectra were subsequently measured to monitor the evolution of TM-O bonds related to the superficial lattice state of LCO. The peaks at 486 and 596 cm^−1^ are ascribed to the bending of O–Co–O bond (*E*_g_) and the stretching of Co–O bond (*A*_1g_) of LCO cathode [37, 38], respectively. During the charging process, the *E*_g_ and *A*_1g_ peaks of LCO cycled in TFE and EE electrolytes both show a decreasing trend in intensity (Fig. 3c). Upon discharging, the peak intensity of the cathode in EE electrolyte is evidently weakened compared with the initial state (Fig. 3c, left), hinting an incomplete lithiation of the electrode triggered by structural damage. On the contrary, the *E*_g_ and *A*_1g_ peaks are fully recovered in TFE electrolyte (Fig. 3c, right). Figure S10 shows the optical images of cycled electrodes in two different electrolytes. The color of the lithium metal surface in EE electrolyte (Fig. S10a) becomes yellowish brown after 20 cycles, indicative of a serious Co dissolution phenomenon. By contrast, the lithium metal still keeps lustrous in TFE electrolyte (Fig. S10b). The Co dissolution amount of LCO after 20 cycles was measured by inductively coupled plasma atomic emission spectrometer (ICP-AES). As depicted in Fig. S11, the dissolved Co amount (0.13 ppm) in TFE electrolyte is much lower than that (1.67 ppm) in EE electrolyte.

Afterward, SEM, HR-TEM and FIB-SEM are employed to intuitively survey the differences in morphology and microstructure evolution of LCO cathodes in different electrolytes. Obviously, the surface structure of cycled LCO in EE electrolyte is severely destroyed and becomes loose and porous (Fig. S12), while the cycled LCO in TFE electrolyte could still maintain its surface and structural integrity. Figure 3d, e shows the cross-sectional images of a large part of the electrode. As shown in Fig. 3d, the cycled LCO in EE electrolyte has numerous cracks within the bulk electrode, which should be ascribed to the substantial internal stress caused by malignant phase transition at high voltage. By comparison, the cycled LCO electrode in TFE electrolyte still remained intact without any fragmentation (Fig. 3e). Figure 3f displays the HR-TEM pattern of the LCO particle cycled in EE electrolyte. As can be clearly seen, most regions of the particle undergo irreversibly transformation from layered structure to spinel/rock-salt phase. By comparison the LCO cathode in TFE electrolyte well maintains its original layered structure with negligible phase transformation (Fig. 3g). The FFT patterns of the selected region in the inset further corroborate the structural evolutions. The above results demonstrate that the elaborate CEI film can effectively inhibit the irreversible phase transition, strengthen the Co–O bond, alleviate the Co dissolution, and eventually enhance the structural and electrochemical stability of LCO cathode (Fig. 3h).

### Enhanced Electrochemical Performance of HV-LCO

Prior to the electrochemical behavior assessments, the electrochemical stability window of both electrolytes is measured by linear sweep voltammetry (LSV) using Li||Al cell. As shown in Fig. S13, the oxidation resistance of TFE electrolyte (4.96 V) is greatly improved compared with that of EE electrolyte (4.35 V), so it can meet the normal working requirements of high-voltage cathode.

**Fig. 4:**
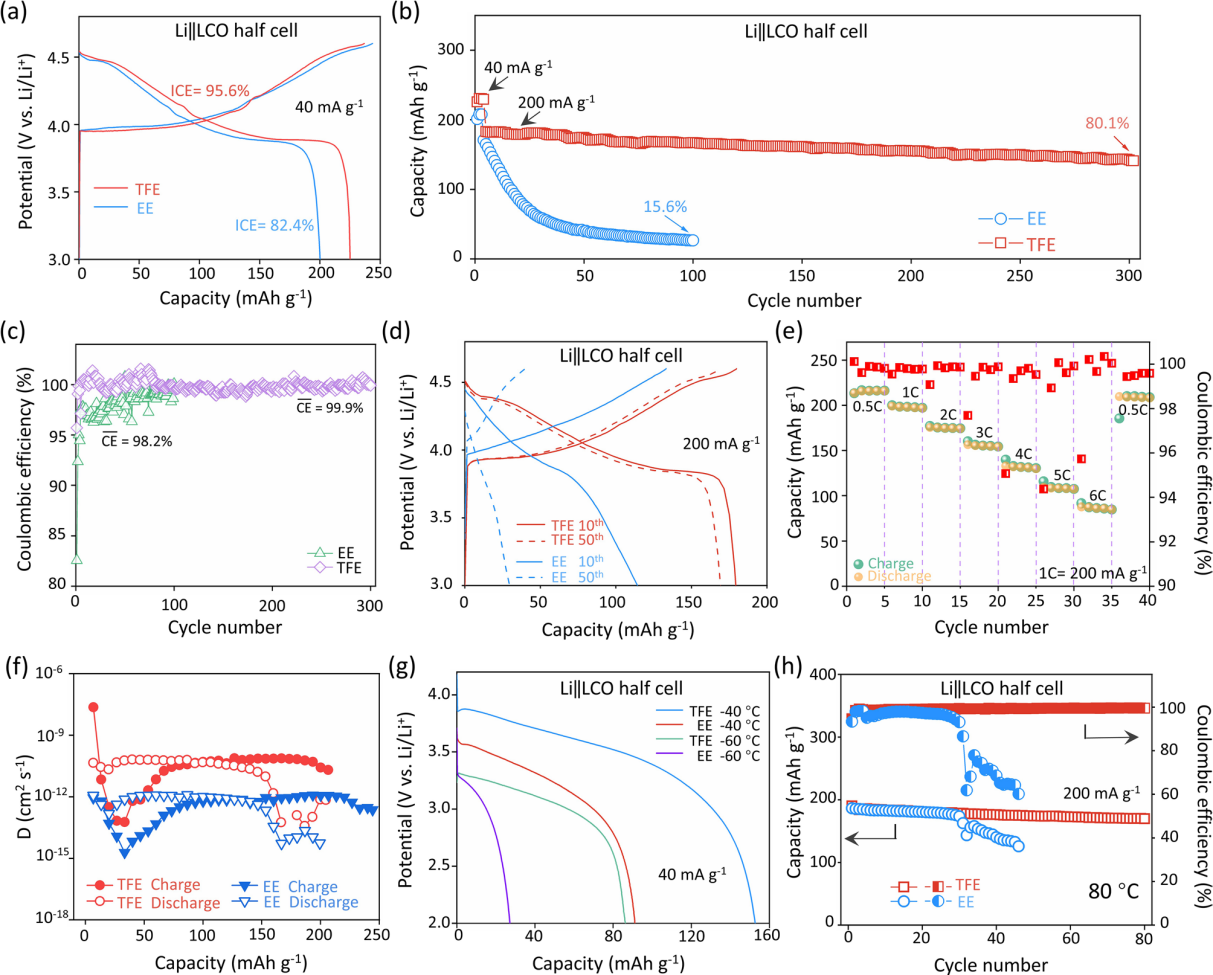
Electrochemical performance of Li||LCO half-cells with different electrolytes. **a** Initial charge/discharge curves of Li||LCO half-cells with different electrolytes; **b**, **c** cycling performance and Coulombic efficiency of Li||LCO half-cells with EE and TFE electrolytes. The specific current density is 40 mA g^−1^ in the first three cycles and 200 mA g^−1^ in the subsequent cycles. The voltage range is 3.0–4.6 V (vs. Li/Li^+^); **d** charge/discharge curves of LCO cathodes in EE and TFE electrolytes at different cycles; **e** rate performance of Li||LCO half-cells with TFE, and cell was charged and discharged at same current; **f** diffusion coefficients of Li||LCO half-cells with EE and TFE electrolytes; **g** low-temperature performance of LCO cathodes in different electrolytes; **h** cycling performance of Li||LCO cells using EE and TFE electrolytes under the current density of 200 mA g^−1^ at 80 °C

Figure 4a–d shows the electrochemical performance of Li||LCO half-cells using different electrolytes between 3.0 and 4.6 V. The half-cell with EE electrolyte in Fig. 4a only offers a reversible specific capacity of 201.0 mAh g^−1^ with an initial Coulombic efficiency (ICE) of 82.4% at a current density of 0.2C (1C = 200 mA g^−1^). Notably, the reversible capacity and ICE are dramatically enhanced to 225.7 mAh g^−1^ and 95.7%, respectively, in TFE electrolyte, which is beneficial for high-energy storage density and energy conversion efficiency of LCO-based batteries. Figure 4b reveals that the capacity retention of the cell using EE electrolyte rapidly drops to 15.5% (compared with the fourth cycle) after only 100 cycles at 1C, and the average Coulombic efficiency (CE) along cycling is 98.2% (Fig. 4c). By contrast, the capacity retention is 80.1% after 300 cycles in TFE electrolyte, while the average CE is as high as 99.9%. The typical galvanostatic charge–discharge curves in Fig. 4d indicate the working voltage of the cell with TFE electrolyte keeps stable, whereas the voltage plateau of the cell with EE electrolyte vanishes rapidly, implying that the LCO structure was seriously damaged. The structural stability of LCO was further identified by the *dq*/*dV* curves during long-term cycles in 3–4.6 V (Fig. S14). The peaks located at 3.85 and 4.45 V correspond to the O3/O3' and O3'/H1-3 phase transitions, respectively. For EE-LCO, both the O3/O3' and O3'/H1-3 phase transitions were reduced upon cycling, indicating the irreversible phase transition and prominent structural degradation. Conversely, TFE-LCO shows weakened O3'/H1-3 phase transition and highly reversible phase transition upon cycles, mainly attributing to the improved LiF-rich CEI layer and well-maintained surface/bulk structure.

Considering the above-mentioned TTE was widely used in high-voltage electrolyte due to its higher anodic stability, 1 mol L^−1^ LiPF_6_-TTE/EMC (3:7 by vol., denoted as TE) is also designed as a comparison. Figure S15 shows that the formed CEI layer is denser and more uniform than that in EE electrolyte; therefore, the cycling performance is better than that of the cell with EE. In spite of this, its performance is still far worse than that with TFE electrolyte, implying the important role of LAE factor in screening suitable solvents.

Besides, to illuminate the role of HOMO in the solvent selection, TEP with similar LAE to TFEP but with unfavorable HOMO is chosen, and 1 mol L^−1^ LiPF_6_-TEP/EMC (3:7, by vol. named as TEE) was prepared for the comparative experiment. The electrochemical performance of Li||LCO half-cell with TEE electrolyte was then tested. As seen in Fig. S16, the initial Coulombic efficiency (ICE) is only 71.3%, and the average CE during cycling is lower than 95%, which should be attributed to the continuous decomposition of TEE at higher voltages. As expected, such a low CE will inevitably lead to poor cycling performance. The above results show that the solvent with high LAE but unfavorable HOMO still cannot meet the demand for high-voltage operation, thereby further demonstrating that both LAE and HOMO are important factors for building robust interface.

The rate performance of LCO cathodes with EE and TFE electrolyte is shown in Figs. 4e and S17; the half-cell was charged and discharged at identical current densities. Clearly, the EE-LCO cathode encountered severe capacity failure only at 2C (0.4 A g^−1^). Sharply reversely, even though with a high loading, the LCO cathode in TFE electrolyte still exhibited outstanding rate capability. A capacity of over 110 mAh g^−1^ was obtained at a high current rate of 5C (1 A g^−1^). When the current returned to 0.5C, the capacity of LCO was fully recovered (~ 210 mAh g^−1^), suggesting a splendid resistance of the thick electrode to the large current shock. The galvanostatic intermittent titration technique (GITT) measurement in Figs. S18 and 4f reveals that the Li^+^ ion diffusion coefficient of LCO in TFE was basically located within the range of 10^−10^–10^−13^ cm^2^ s^−1^, which is one order of magnitude higher than that in EE electrolyte. Two factors might have contribution to the superior rate capability of the Li||LCO cell with TFE electrolyte. First, the thin and uniform TFEP-derived CEI film enables fast Li^+^ transport at the interface. Secondly, the stable structure of LCO cathode in TFE electrolyte can guarantee the rapid diffusion of Li^+^ ions, so as to avoid the blockage of Li^+^ ion diffusion pathway induced by the phase transition of LCO in EE electrolyte.

Improving the wide-temperature operation of LIBs is crucial for advancing their application scope in harsh environments; hence, the temperature-dependent properties of the two electrolytes were characterized. Figure S19 depicts the ionic conductivity of both electrolytes from − 60 to 80 °C. TFE has a slightly lower conductivity than EE above 0 °C, but at low temperature, its conductivity is better than that of EE. This superiority becomes more significant with the decrease of temperature. For instance, the TFE electrolyte still has a conductivity of over 0.2 mS cm^−1^ at − 60 °C, whereas the EE electrolyte has solidified at − 50 °C. Besides, the high boiling point (131 °C) of TFEP also enhances the thermostability of the electrolyte (Fig. S20).

Before the low-temperature tests, all the cells were precycled for 5 cycles and fully charged at room temperature. Then, the coin cells were discharged to 2.0 V with a current density of 40 mA g^−1^ at − 40 and − 60 °C. As shown in Fig. 4g, the cell with TFE electrolyte retains 70% and 40% of its room-temperature capacity at − 40 and − 60 °C, respectively, far exceeding the capacity retention (45% and 15%) of the cell with EE electrolyte at the same temperatures. More significantly, the average working voltage of the cell with TFE electrolyte is still maintained above 3.2 V at − 60 °C, manifesting exceptional low-temperature adaptability. Upon high-temperature test, Fig. 4h shows that the Li||LCO half-cell with EE electrolyte encounters a rapid failure in mere 30 cycles at 80 °C, whereas the cell with TFE electrolyte exhibits appreciable cycling stability (> 90% capacity retention after 80 cycles).

### Ah-Grade Gr||LCO Pouch Cells and Safety Tests

The benefits of TFE electrolyte to HV-LCO cathode are further assessed in lithium-ion full-cell. Before that, the compatibility between TFE and graphite (Gr) anode is explored. The Li||Gr half-cell with TFE electrolyte delivers a favorable reversible capacity of 355.3 mAh g^−1^ with an ICE of 87.8% in the first cycle (Fig. S21). Also, the half-cell exhibits negligible capacity decline after 250 cycles (99.4% capacity retention), thereby offering a desirable anode material for the construction of HV-LCO-based full-cell. To investigate the root cause for the great electrochemical compatibility, we also calculated the adsorption energy of the solvent on the surface of graphite anode, and the absorption model is shown in Fig. S22. Similar to LAE, among the 10 typical flame-retardant solvents and common carbonate solvents, TEP and fluorinated derived solvents (FTEP, TFEP) exhibited the most prominent carbon absorption energy (CAE, Fig. S23a), indicating that TFEP would preferentially absorb on the graphite anode. Besides, the low LUMO energy level of TFEP implies its reduction tendency on Gr surface (Fig. S23b) and will boost the construction of F-rich SEI film, like its evolution behavior on LCO cathode.

**Fig. 5:**
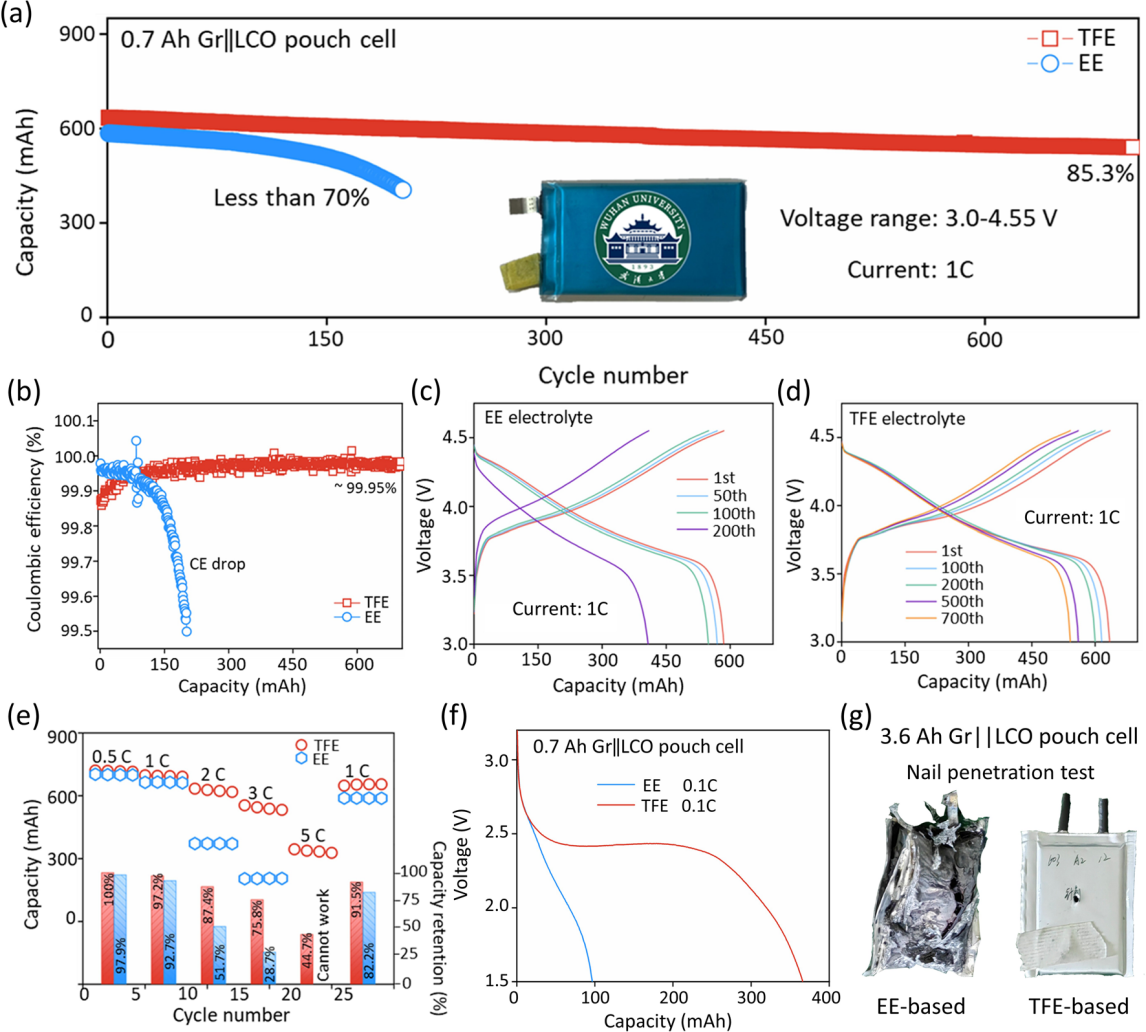
Electrochemical performance and safety tests of Gr||LCO pouch cell with TFE. **a**, **b** Cycling performance and Coulombic efficiencies of 0.7 Ah Gr||LCO pouch cells with EE and TFE electrolytes. The current is 700 mA and the voltage range is 3.0–4.55 V; **c**, **d** galvanostatic charge/discharge profiles of pouch cells using EE and TFE electrolytes with different cycles at 1C (1C = 700 mA); **e** rate performance of pouch cells with different electrolytes. The cells are charged at 0.2C and discharged at corresponding current; **f** discharge curves of 0.7 Ah Gr||LCO pouch cells with different electrolytes at − 60 °C under the current of 0.1C. **g** Optical photographs of 3.6 Ah pouch cell with EE and TFE electrolytes (fully charged) after the nail penetration tests

Based on the above HV-LCO and graphite anode, 0.7 Ah Gr||LCO pouch cells with two different electrolytes were assembled. Figure 5a, b shows the cycling performance and the corresponding Coulombic efficiency at 1C in the voltage range of 3.0–4.55 V. The pouch cell with TFE attains high capacity retention of 85.3% after 700 cycles with average CE over 99.95% (Fig. 5b). By comparison, both the capacity and CE of the cell with EE electrolyte decline rapidly, and only 69.2% of its initial capacity can be reserved after 200 cycles. The typical charge–discharge curves in Fig. 5c, d indicate the stable operating voltage of the pouch cell with TFE electrolyte. Figure 5e compares the rate performance of both cells. The cell with TFE retains 75.8% and 44.7% of its initial capacity at 3C and 5C, respectively, while the cell with EE has almost no capacity at 5C. More importantly, it can be seen that the working voltage of the cell with TFE electrolyte maintained above 3.41 V at 5C, which is still higher than that of the cell with EE electrolyte (3.39 V) at 3C (Fig. S24). In addition to the rate performance tests, the low-temperature performance of both pouch cells is also measured, and the cells were fully charged and then discharged to 1.5 V with a current of 70 mA at − 60 °C. Figure 5f reveals that the cell with TFE electrolyte delivers an inspiringly capacity of 0.37 Ah (> 52.9% capacity retention) and an average operating voltage of 2.43 V at − 60 °C, which is far superior to that (0.1 Ah, less than 15% capacity retention rate) of the cell with EE electrolyte.

As mentioned above, the high oxidation state of Co^4+^ in charged HV-LCO catalyzes the decomposition of electrolyte, which will cause premature risk at high voltage. In addition, the high flammability of commercial carbonate solvents will aggravate the safety hazards. Encouragingly, the flame-retardant TFEP may promote the safety of the HV-LCO-based batteries. First of all, the flammability of both electrolytes is roundly evaluated by ignition tests. As shown in Fig. S25, the membrane soaked in EE electrolyte immediately combusts, significantly different from the TFE-soaked membrane. Whereafter, fully charged 3.6 Ah Gr||LCO pouch cells (the energy density at 0.2C reaches 261.1 Wh kg^−1^) are assembled for nail penetration test (considered as the most stringent safety test, the corresponding cycling performance of the pouch cells is shown in Fig. S26). Figures 5g and S27 display the optical images of the pouch cells with different electrolytes after nail penetration test and time–voltage/temperature curves during tests. The cell with EE electrolyte suffers a severe explosion, while the cell with TFE electrolyte remains intact without any deformation. Besides, the voltage of cell using EE electrolyte dropped to 0 V immediately upon nail penetration and induced thermal runaway (over 450 °C). Encouragingly, the pouch cell with TFE electrolyte underwent only a slight voltage drop of ~ 0.05 V. Besides, the cell temperature maintained below 50 °C for 1 h during nail penetration. The results above demonstrated that TFE electrolyte can enhance the safety of high-voltage LCO-based LIBs. In summary, TFE electrolyte endows Gr||LCO Ah-grade pouch cell excellent cycling performance, wide operating temperature and enhanced safety, which exceeds many other high-voltage LCO cathodes reported so far [39–49] (Fig. S28).

### General Applicability of LAE-HOMO Descriptor

To validate the universality of this high-voltage electrolyte design principle, we then extended the application of TFEP-based electrolyte to NMC811 cathode in LIBs and Na_2/3_Ni_1/3_Mn_2/3_O_2_ cathode in SIBs. Figure S29a, b displays the cycling performance of Li||NMC811 half-cells with TFE and EE electrolytes in the voltage range of 3.0–4.6 V. As can be seen, the half-cell with TFE exhibited capacity retention of 91.0% after 100 cycles with an average Coulombic efficiency of 99.5%, both of which are far superior to those of half-cell with EE. Besides, the working potential of the cell with TFE kept unchanged during cycling. In sharp contrast, the working potential of the cell with EE declined rapidly, suggesting that the structure of NMC811 was completely damaged. Even in sodium-ion batteries systems, this principle also throws great effect. The superiority of TFE electrolyte was also evidenced in sodium-ion batteries. The Na||Na_2/3_Ni_1/3_Mn_2/3_O_2_ half-cell (2.0-4.0 V, vs. Na/Na^+^) with 1 mol L^−1^ NaPF_6_-TFEP/EMC (3:7, by vol.) offered notably enhanced cycling performance and working voltage retention compared with the cell with commercial EC-based electrolyte (Fig. S29c, d), confirming the general applicability of LAE-HOMO descriptor in improving high-voltage electrochemical stability.

Additionally, aiming at improving the safety of high-voltage LCO-based battery, the solvents assessed in this work should possess certain flame-retardant properties, which limit the choice of available solvents. Hence, we evaluated more representative solvents that have been reported in previous works (including carbonate, nitrile, ether, sulfone, amide). As shown in Fig. S30, TMS and AN exhibit similar HOMO energy level to fluorinated solvents, indicating that they have favorable oxidation resistance. In terms of LAE, although several solvents have higher LAE value than TFEP, their poor HOMO energy level is far from the high-voltage demand. Hence, AN is the optimal choice for high-voltage LCO cathode regardless of safety. In fact, AN has been recognized as an effective high-voltage additive in LIBs, but is restricted by its poor compatibility with graphite anode. This result further confirms the reliability of the HOMO-LAE binary solvent descriptor.

## Conclusion

In summary, a novel electrolyte design tactic is proposed to improve the structural stability of HV-LCO, widen the working temperature range and enhance the intrinsic safety. HOMO energy levels and LCO absorption energy are defined as the two key factors to screen available solvents, and TFEP is determined as the optimized one among ten typical flame-retardant solvents. TFEP is identified with much lower defluorination energy barrier than dehydrogenation and thereby benefits the formation of a stable and robust F-rich CEI film, which not only inhibits the irreversible O3/H1-3 phase transition of LCO at high operating voltage, but also enables fast Li^+^ ion diffusion kinetics. As a result, the TFE electrolyte endows HV-LCO with excellent cycling stability (85.3% capacity retention after 700 cycles), rate capability (5C) and wide-temperature adaptability (− 60–80 °C) in both half-cell and full-cell applications. More significantly, the battery safety based on HV-LCO cathode is substantially enhanced (pass the nail penetration test) due to the nonflammable characteristic of TFE. We believe that the high-performance TFE electrolyte can promote the reliable and large-scale applications of 4.6 V LiCoO_2_ in high-energy density LIBs. Also, the novel strategy of manipulating electrolyte chemistry will provide guidance for the rational design of highly compatible electrolyte in rechargeable batteries.

## Supplementary Information

Below is the link to the electronic supplementary material.

Supplementary file1 (DOCX 18684 KB)
