# Supplementary material for: Manipulating Interfacial Stability via Preferential Absorption for Highly Stable and Safe 4.6 V LiCoO2 Cathode
Source: Nanomicro Lett. 2025 Mar 12;17:181. doi: 10.1007/s40820-025-01694-4 (PMC11904031; doi:10.1007/s40820-025-01694-4)
Supplement: Supplementary file 1 — Supplementary file1 (DOCX 18684 KB) [file 40820_2025_1694_MOESM1_ESM.docx]

Supporting Information for

**Manipulating Interfacial Stability via Preferential Absorption for Highly Stable and Safe 4.6 V LiCoO_2_ Cathode**

Long Chen^1#^, Xin He^2#^, Yiqing Chen^1^, Youmin Hou^1^*, Yujie Zhang^2^, Kangli Wang^2^*, Xinping Ai^3^, Yuliang Cao^3^, and Zhongxue Chen^1^*

^1^ Key Laboratory of Hydraulic Machinery Transients, Ministry of Education, School of Power and Mechanical Engineering, Wuhan University, Wuhan 430072, P. R. China

^2^ State Key Laboratory of Advanced Electromagnetic Technology, School of Electrical and Electronic Engineering, Huazhong University of Science and Technology, 430074, Wuhan, P. R. China

^3^ Hubei Key Laboratory of Electrochemical Power Sources, College of Chemistry and Molecular Sciences, Wuhan University, Wuhan 430072, P. R. China

^#^ Long Chen and Xin He contributes equally to this work.

*Corresponding authors. E-mail: [houyoumin@whu.edu.cn](mailto:houyoumin@whu.edu.cn) (Youmin Hou); [zxchen_pmc@whu.edu.cn](mailto:zxchen_pmc@whu.edu.cn) (Zhongxue Chen); [klwang@hust.edu.cn](mailto:klwang@hust.edu.cn) (Kangli Wang)

**Supplementary Figures and Tables**

*
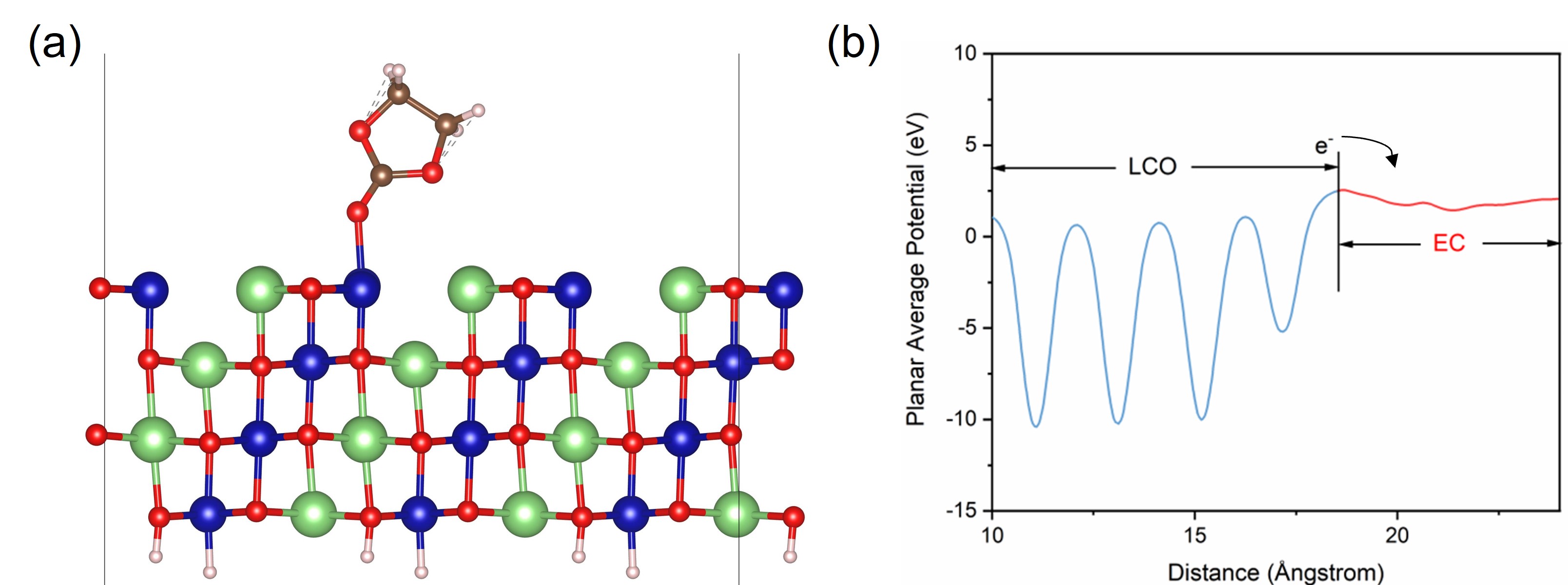
*

**Fig. S1** (**a**) Absorption model of EC-LCO; (**b**) Planar average total potential energy along the Z direction


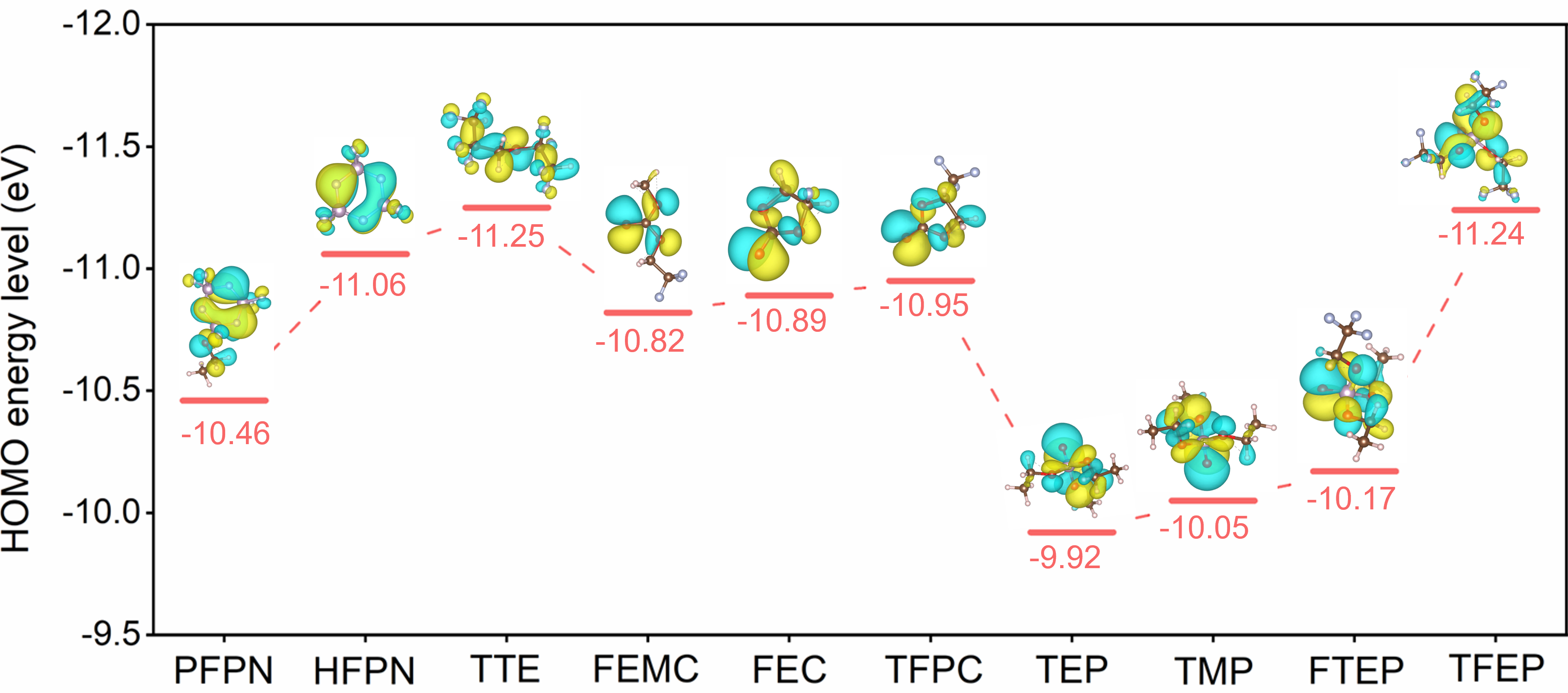


**Fig. S2** HOMO energy levels of ten typical flame-retardant solvents


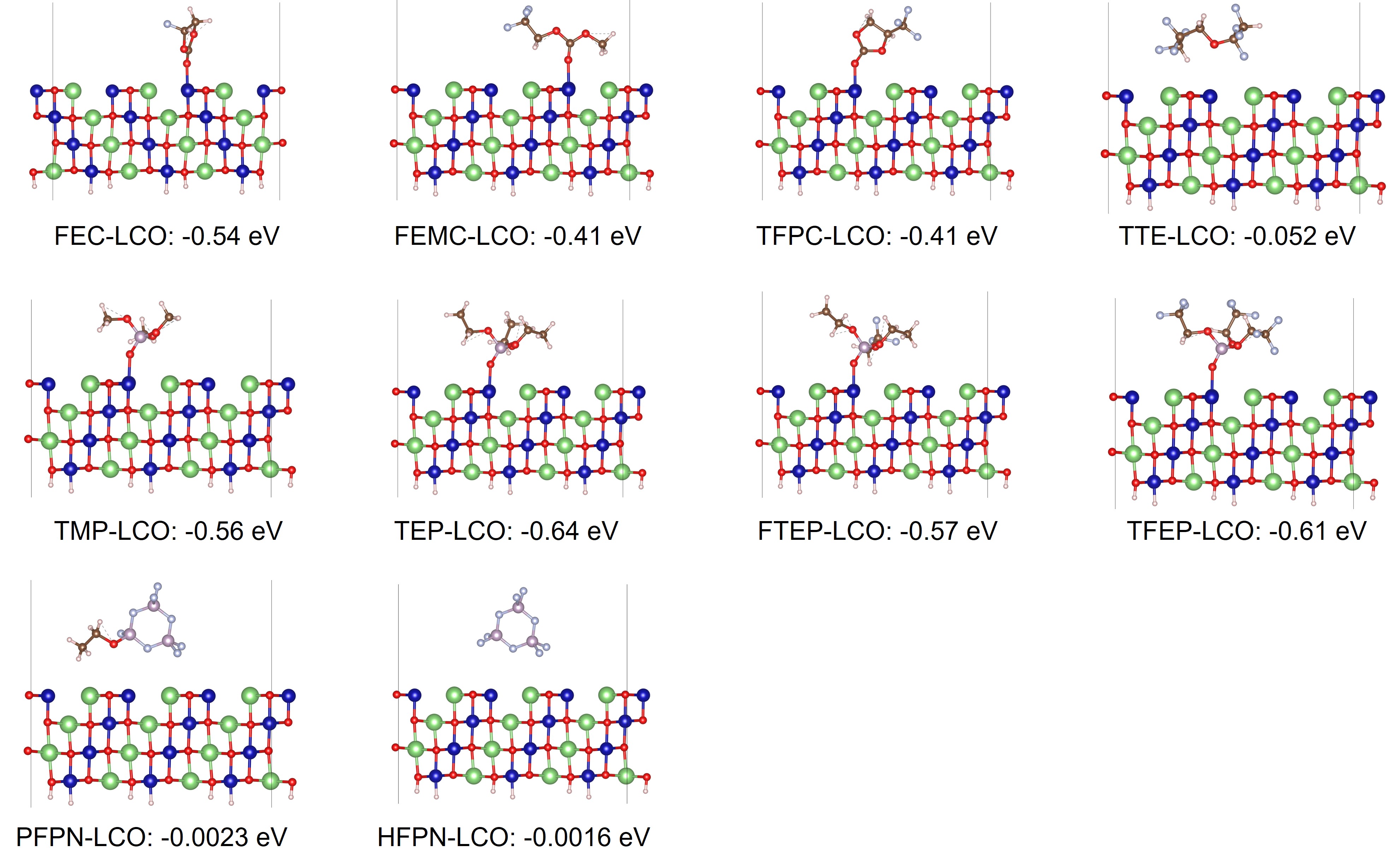


**Fig. S3** The absorption energies and models of ten typical flame-retardant solvents on LCO surface


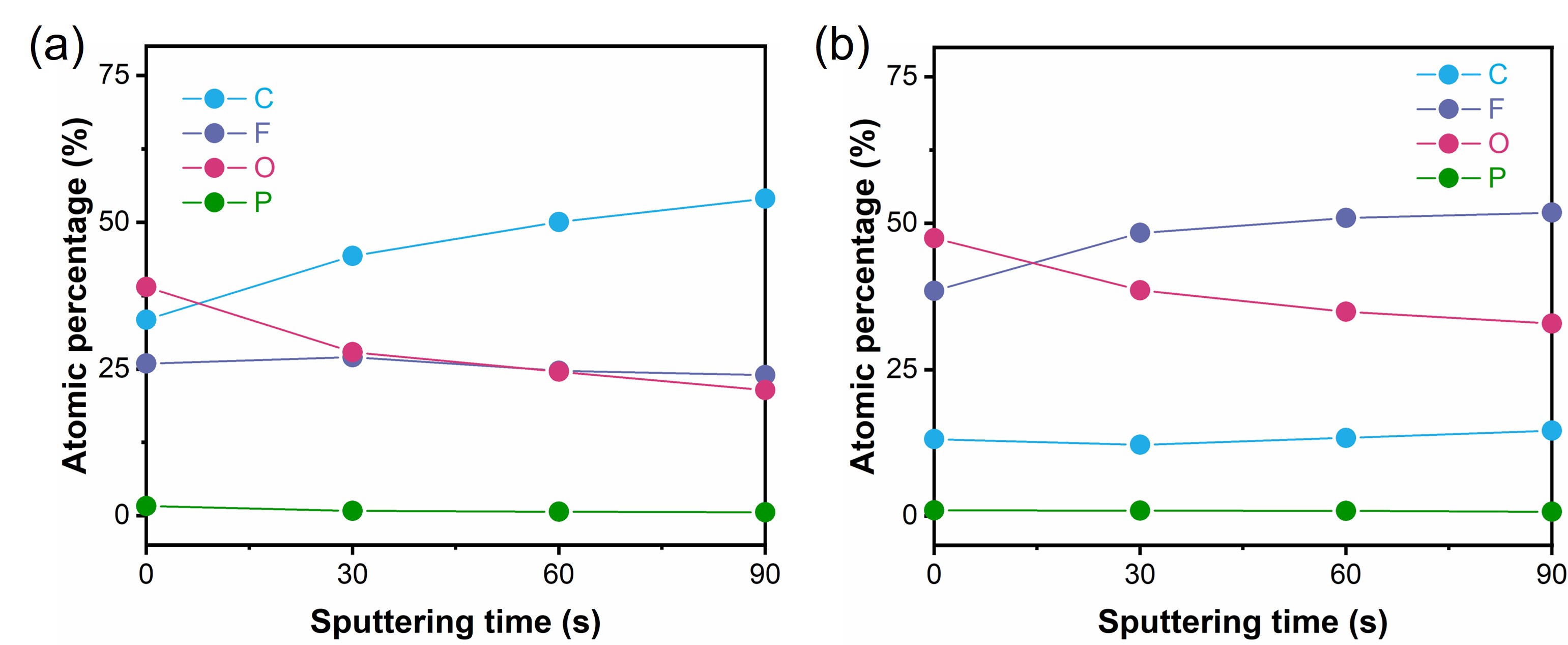


**Fig. S4** Atomic percentage changes of CEI films formed on LCO electrode surface after 20 cycles in (**a**) EE and (**b**) TFE electrolytes with different etching time


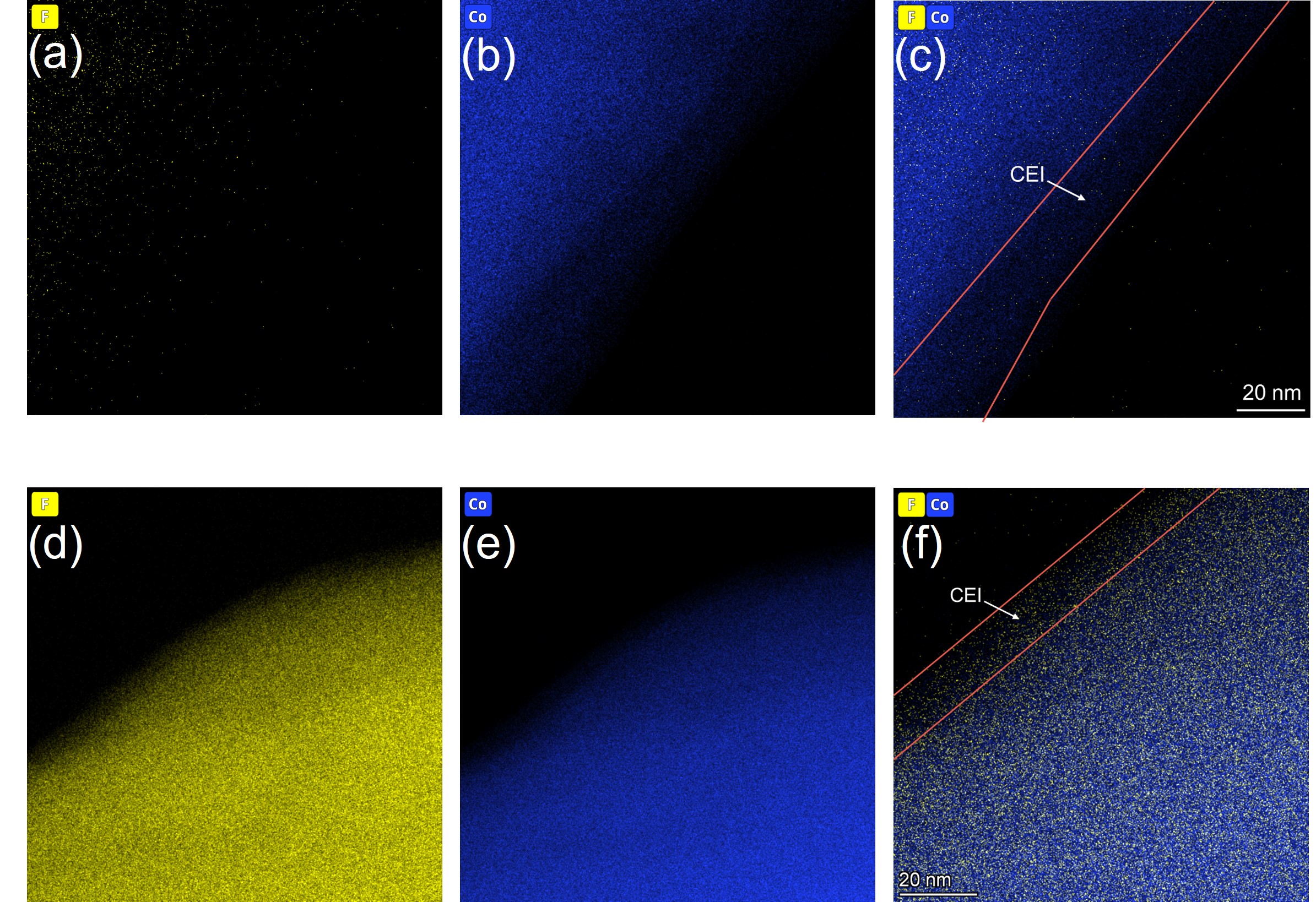


**Fig. S5** The STEM-EDS results of LCO after cycling in (**a-c**) EE and (**d-e**) TFE. Note: Co element (in blue) and F element (in yellow)


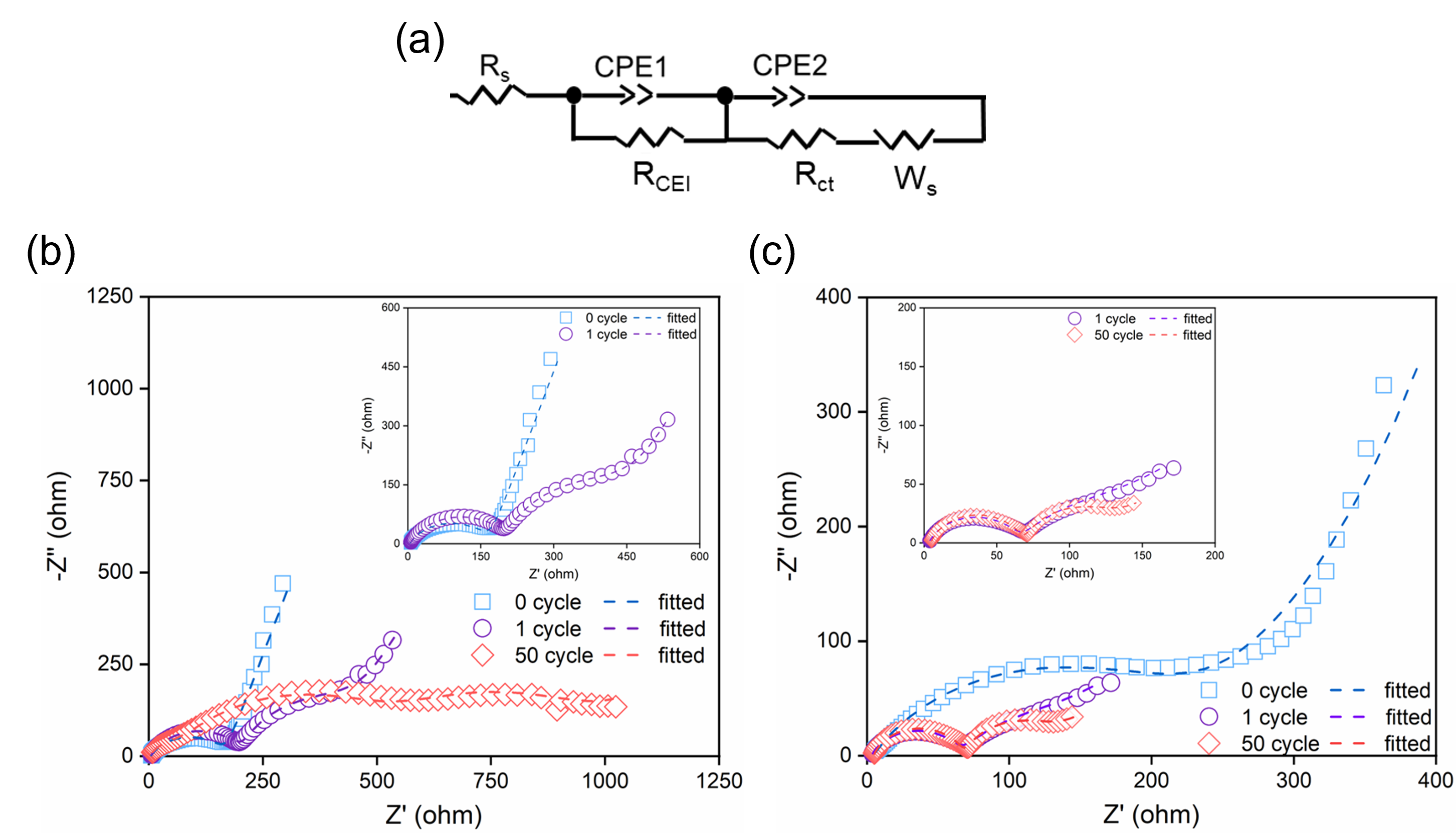


**Fig. S6** (**a**) Equivalent circuit; EIS results of Li/LCO half cells with (**b**) EE electrolyte and (**c**) TFE electrolyte after different cycles


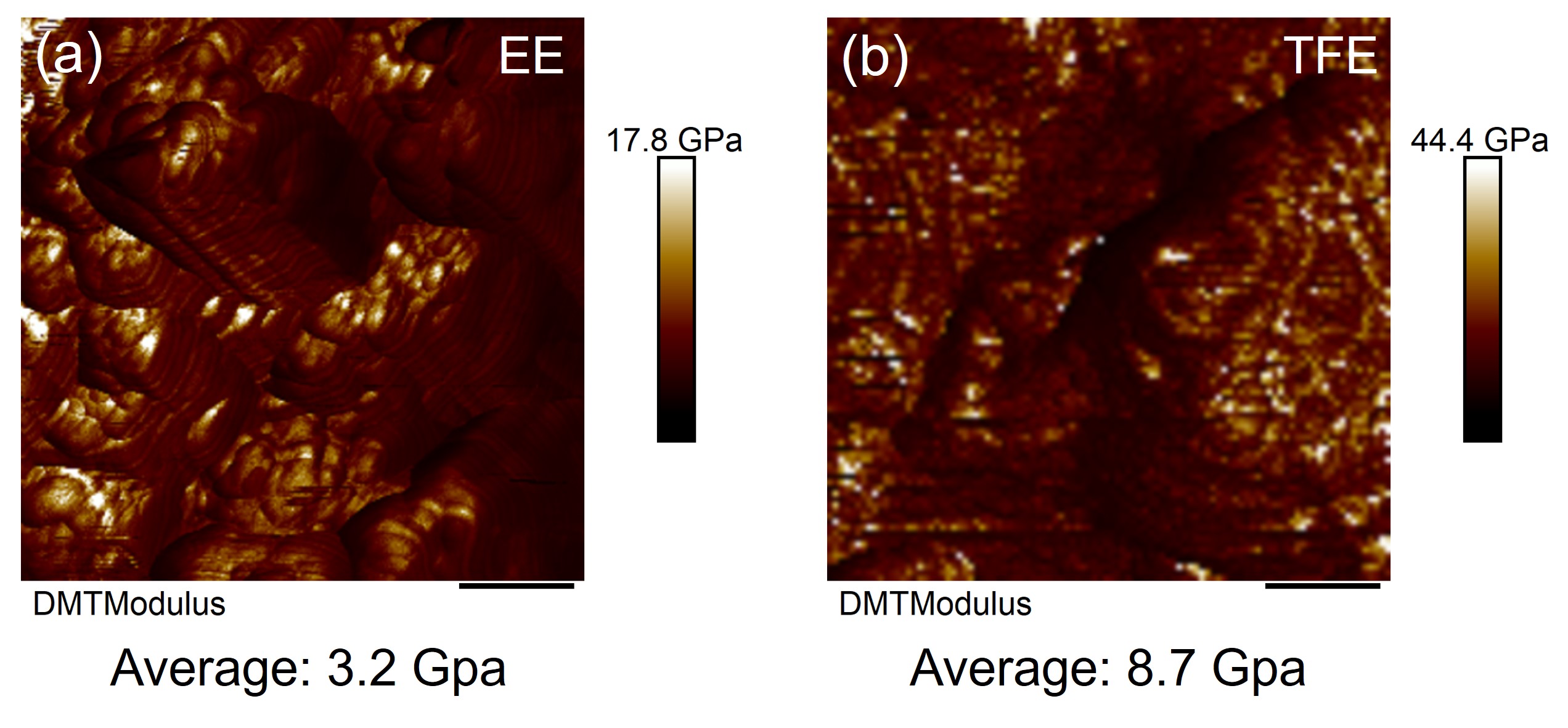


**Fig. S7** The Young’s modulus of CEI film formed in (**a**) EE and (**b**) TFE electrolytes


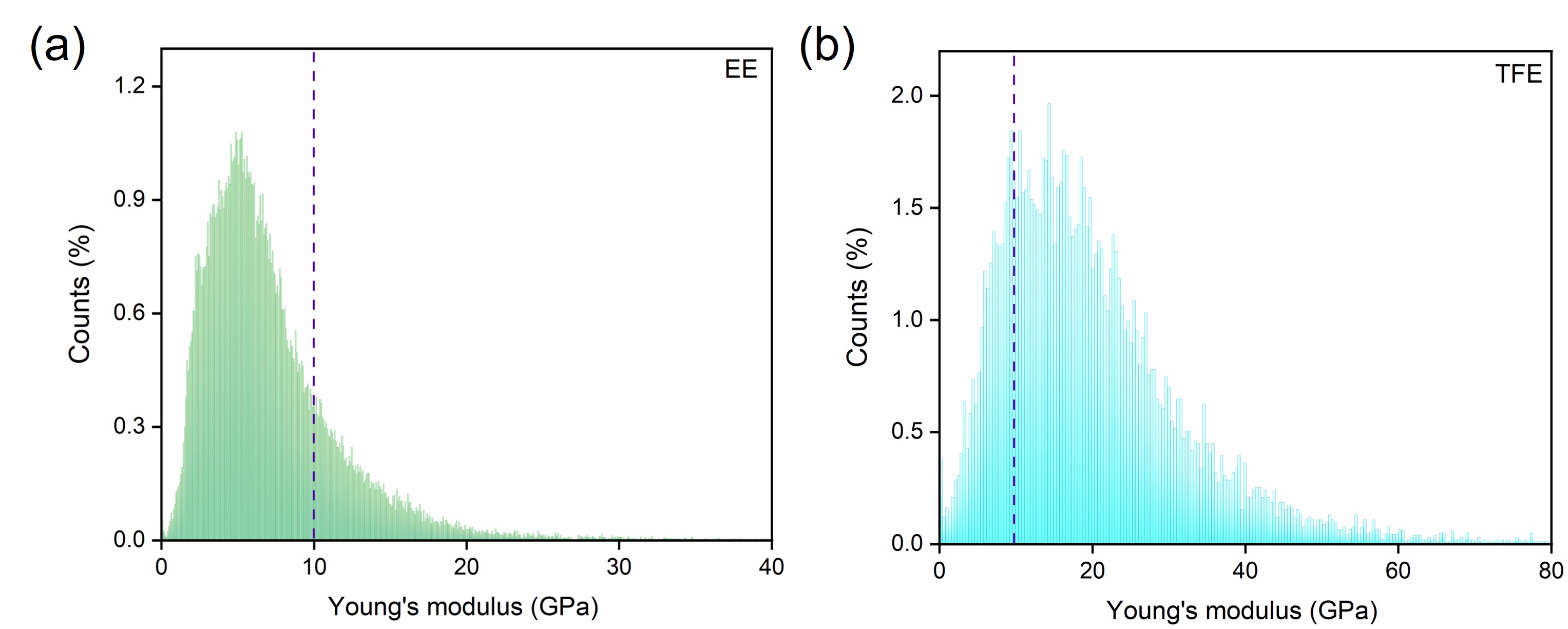


**Fig. S8** The Young’s modulus of CEI film formed in (**a**) EE and (**b**) TFE electrolytes


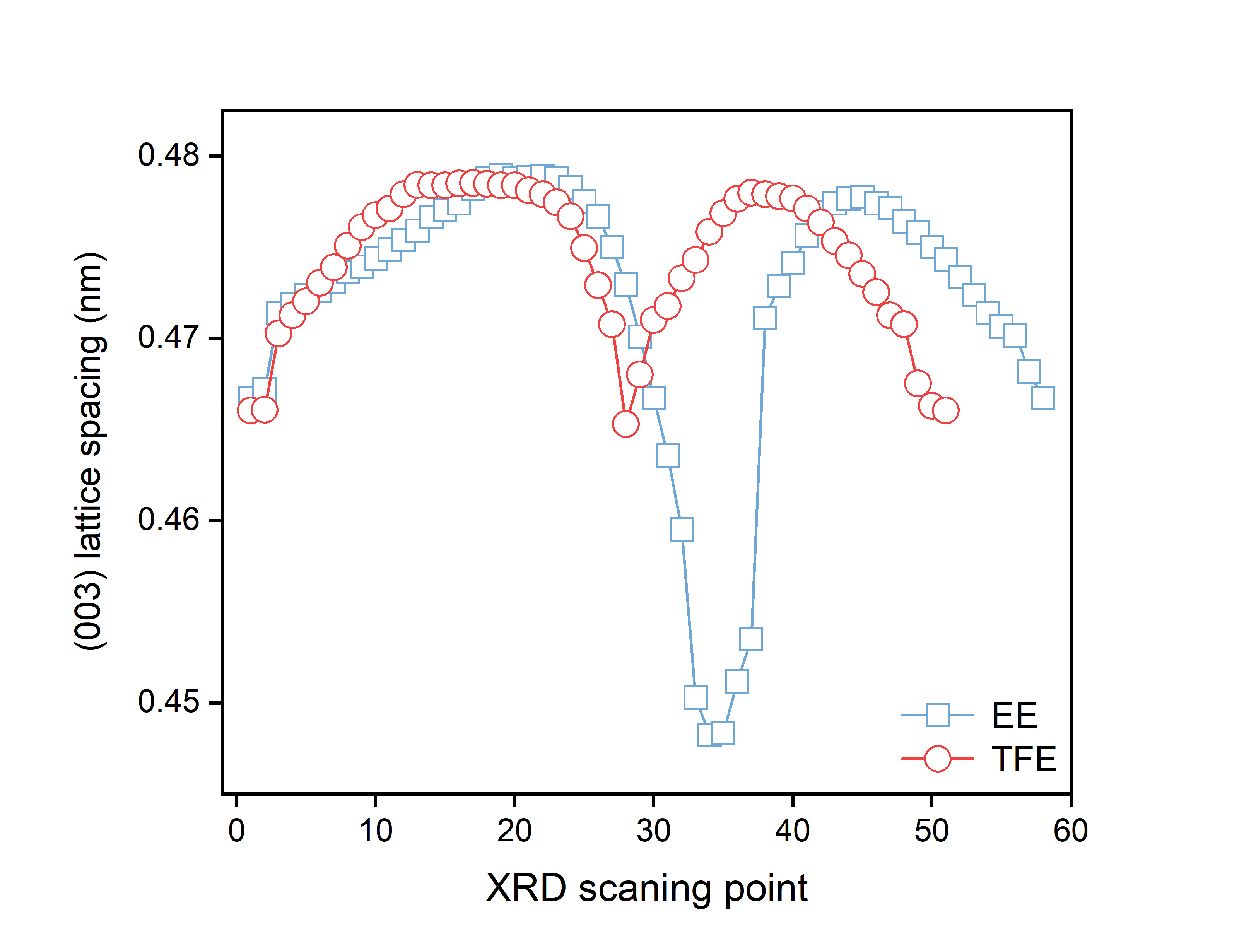


**Fig. S9** The (003) peak lattice spacing of LCO during cycling with different electrolytes


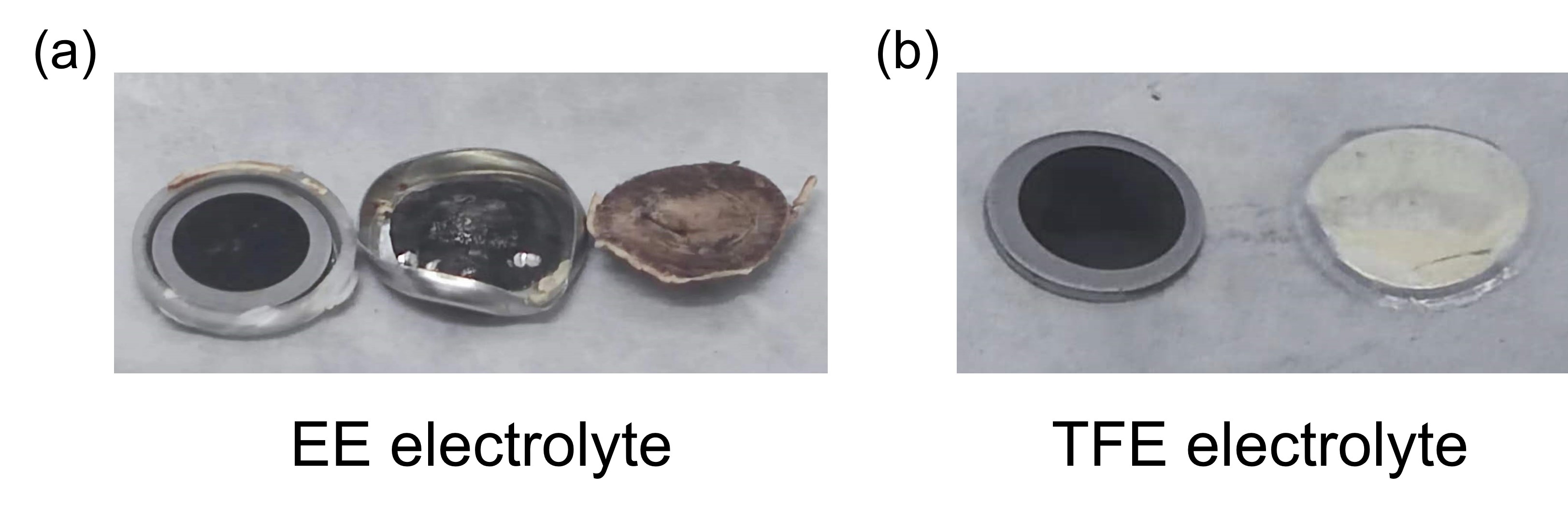


**Fig. S10** Optical photographs of Li electrodes cycling in (**a**) EE and (**b**) TFE electrolytes after 20 cycles


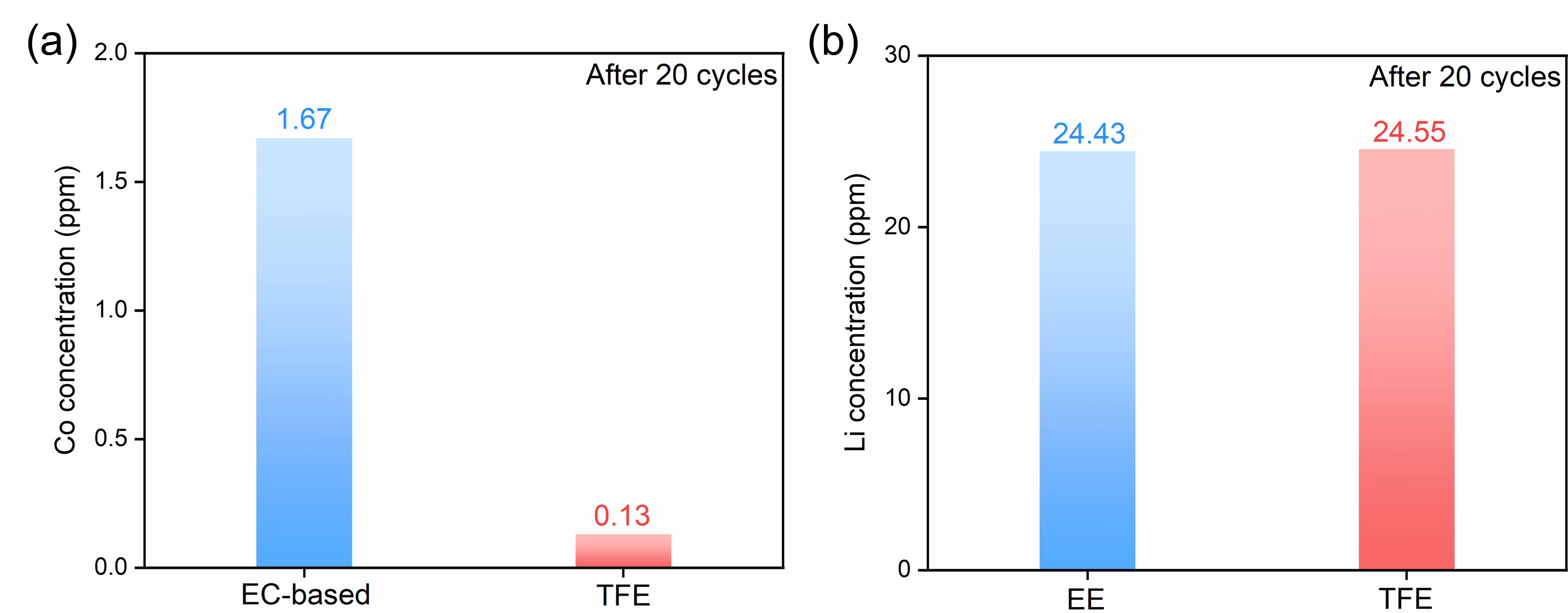


**Fig. S11** The contents of (**a**) Co and (**b**) Li ions in different electrolytes after 20 cycles


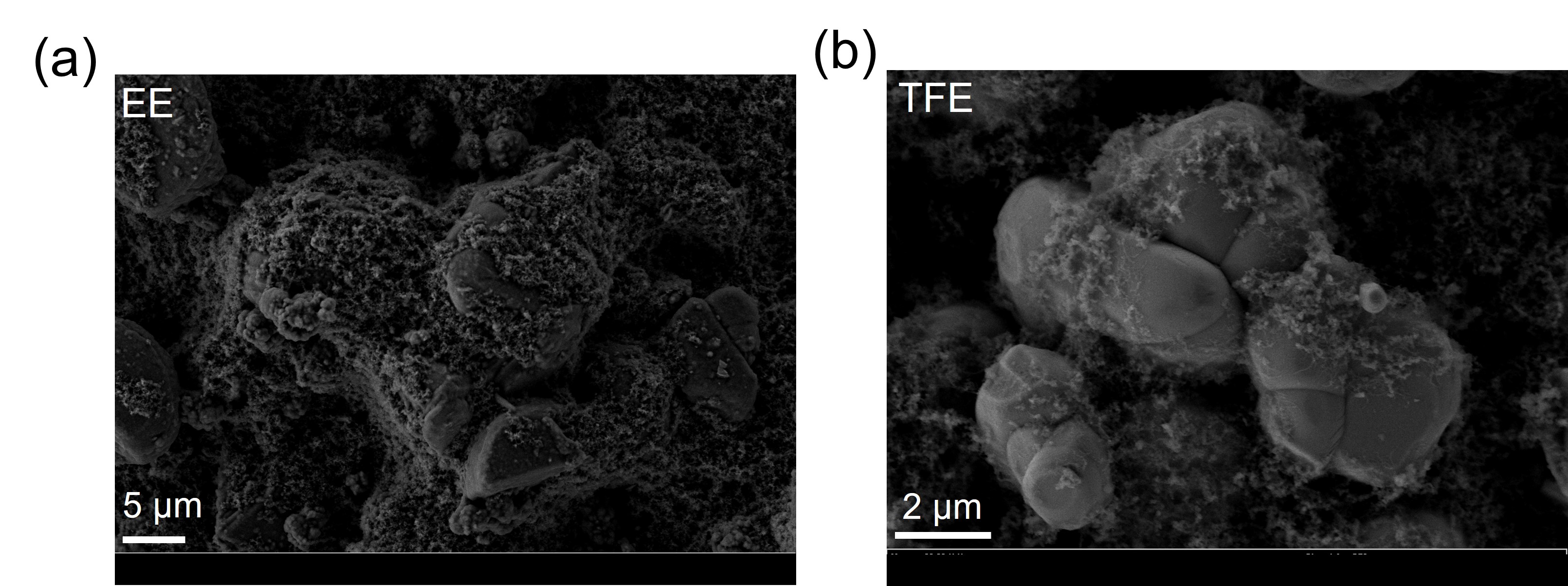


**Fig. S12** SEM images of LCO cathodes cycling in (**a**) EE and (**b**) TFE electrolyte after 20 cycles


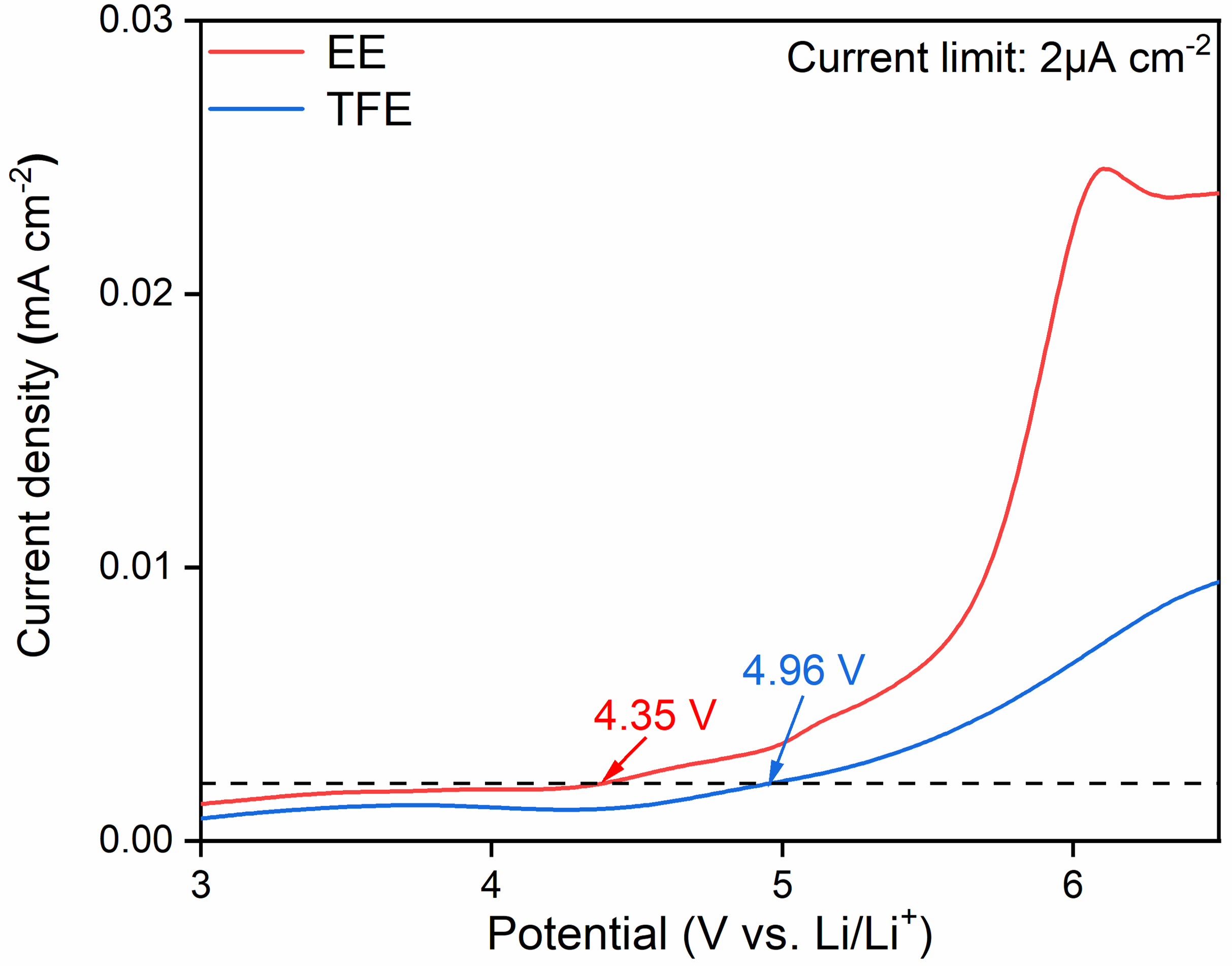


**Fig. S13** Linear sweep voltammetry results of Li||Al half cells with different electrolytes and the scan rate is 10 mV s^-1^


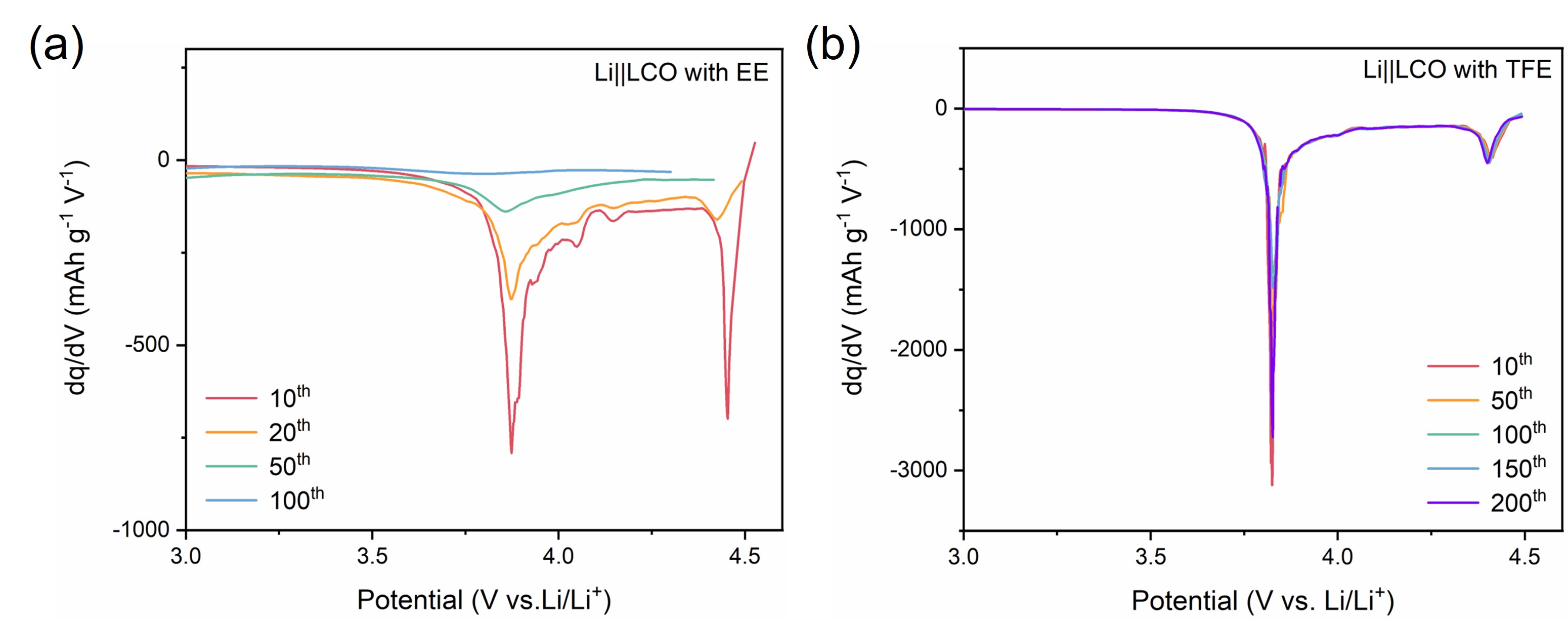


**Fig. S14** dQ/dV curves of Li||LCO cells after different cycles with (**a**) EE and (**b**) TFE electrolyte in a voltage range of 3–4.6 V


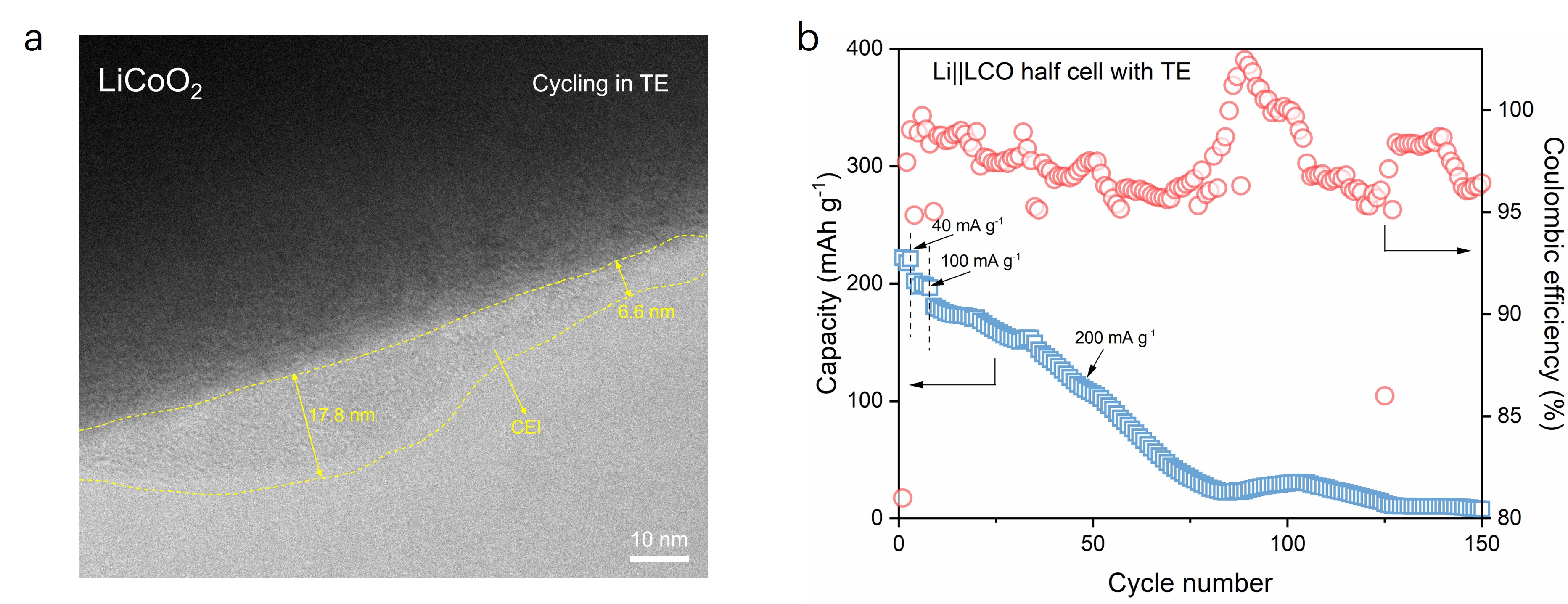


**Fig. S15** (**a**) HR-TEM images of CEI film formed on LCO with TE electrolyte. (**b**) Cycling performance and Coulombic efficiency of Li||LCO half-cell with TE electrolyte under a voltage range from 3.0-4.6 V


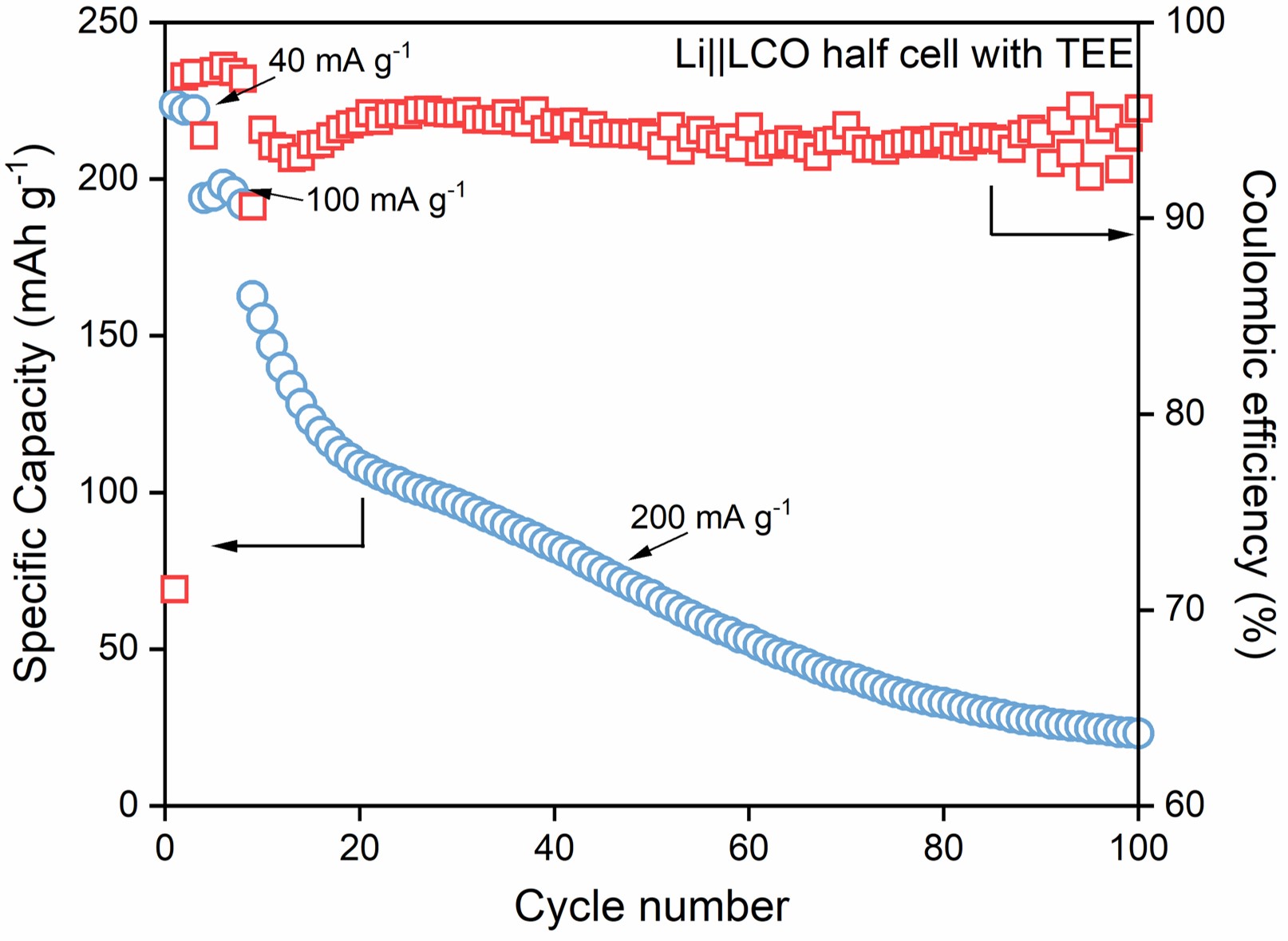


**Fig. S16** Cycling performance and Coulombic efficiency of Li||LCO half-cell with TEE electrolyte (the specific current density is 40 mA g^−1^ for the first three cycles, 100 mA g^−1^ for the following five cycles, and 200 mA g^−1^ for the subsequent cycles)





**Fig. S17** (a) HR-TEM images of CEI film formed on LCO with TE electrolyte. (b) Cycling performance and Coulombic efficiency of Li||LCO half-cell with TE electrolyte under a voltage range from 3.0-4.6 V


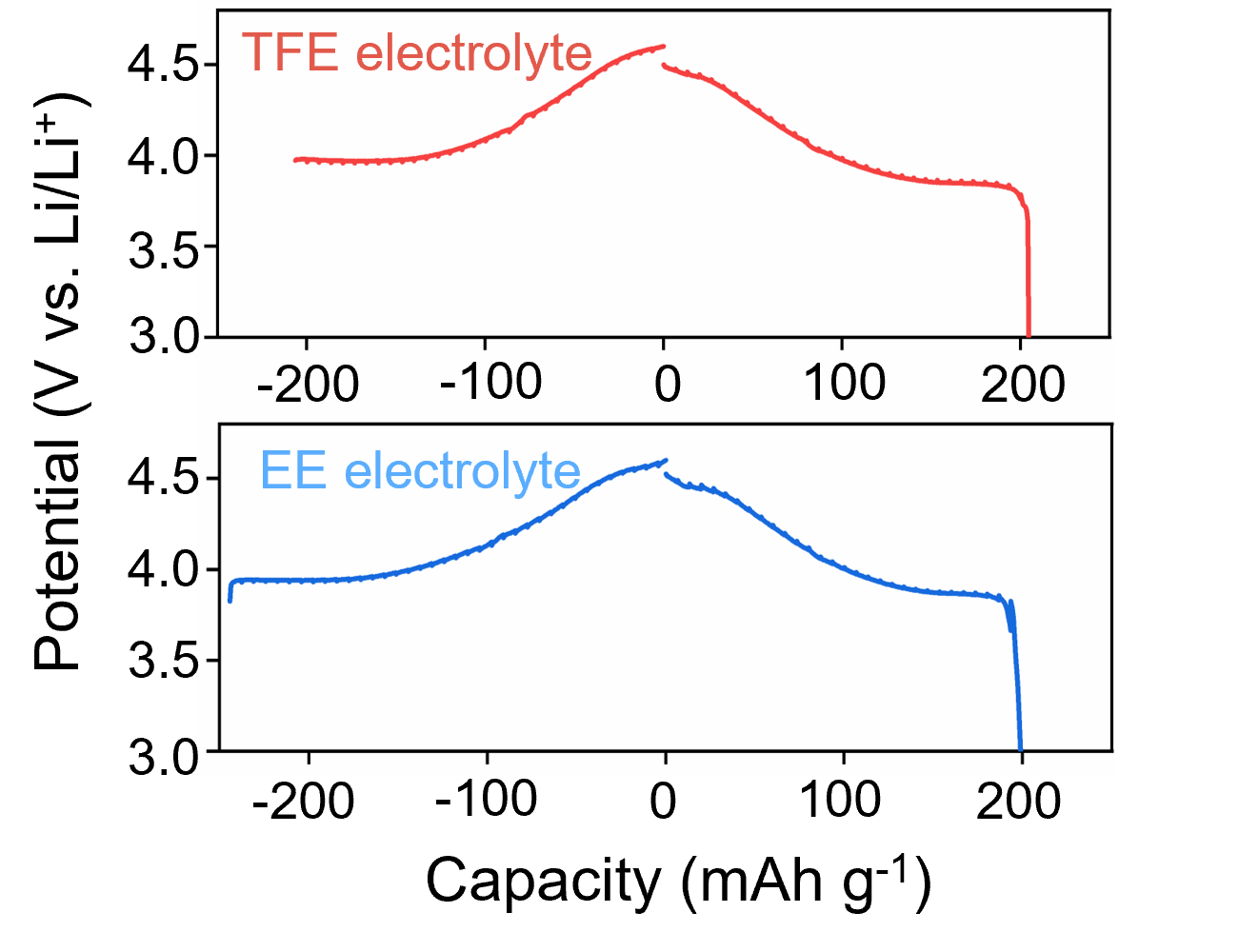


**Fig. S18** GITT curves of Li||LCO half cells with TFE (above) and EE (below) electrolytes





**Fig. S19** The ionic conductivity of different electrolytes from -60 ℃ to 80 ℃


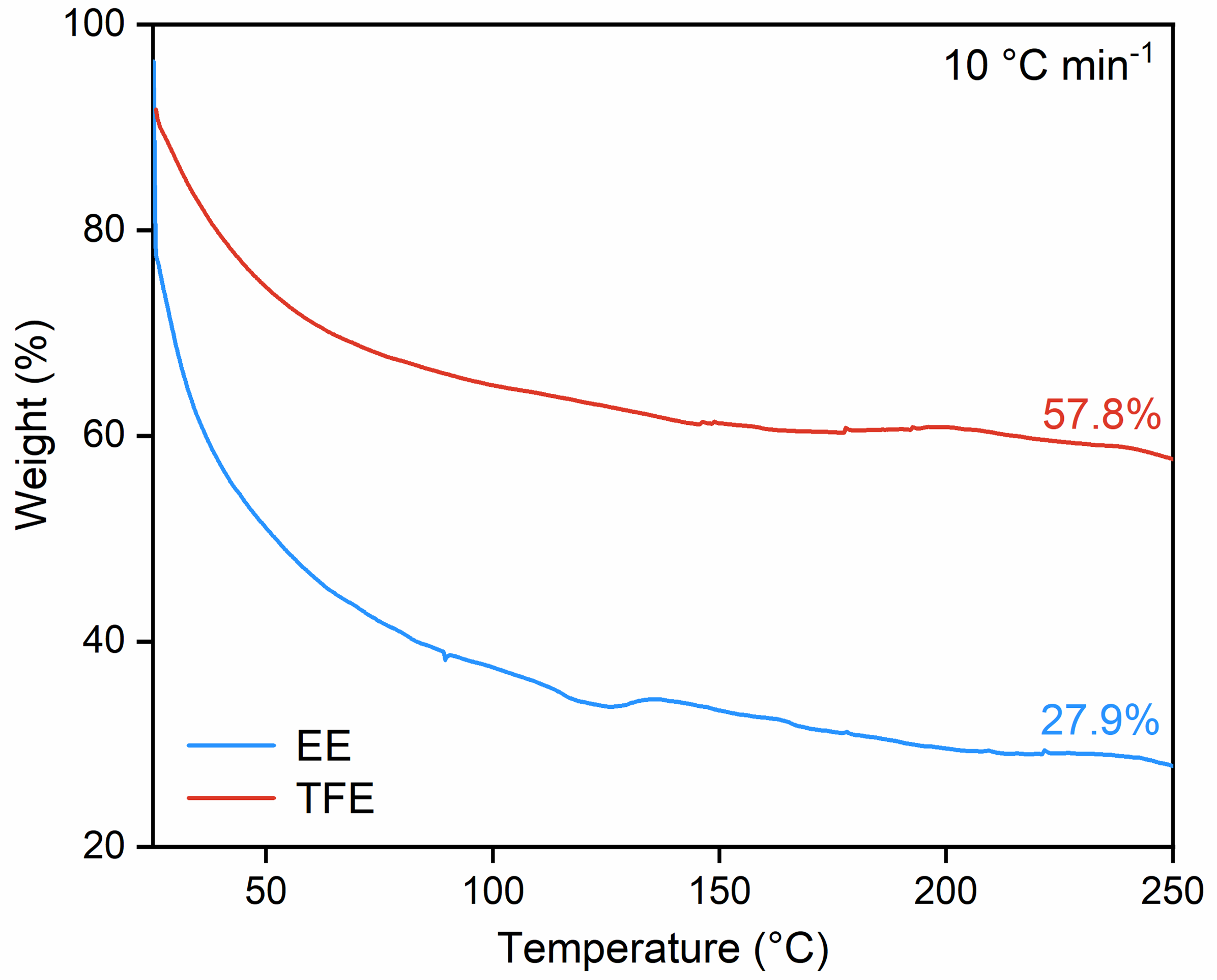


**Fig. S20** TG results of EE and TFE electrolytes from room temperature to 250 ℃, the temperature rising rate was 10 ℃ min^-1^


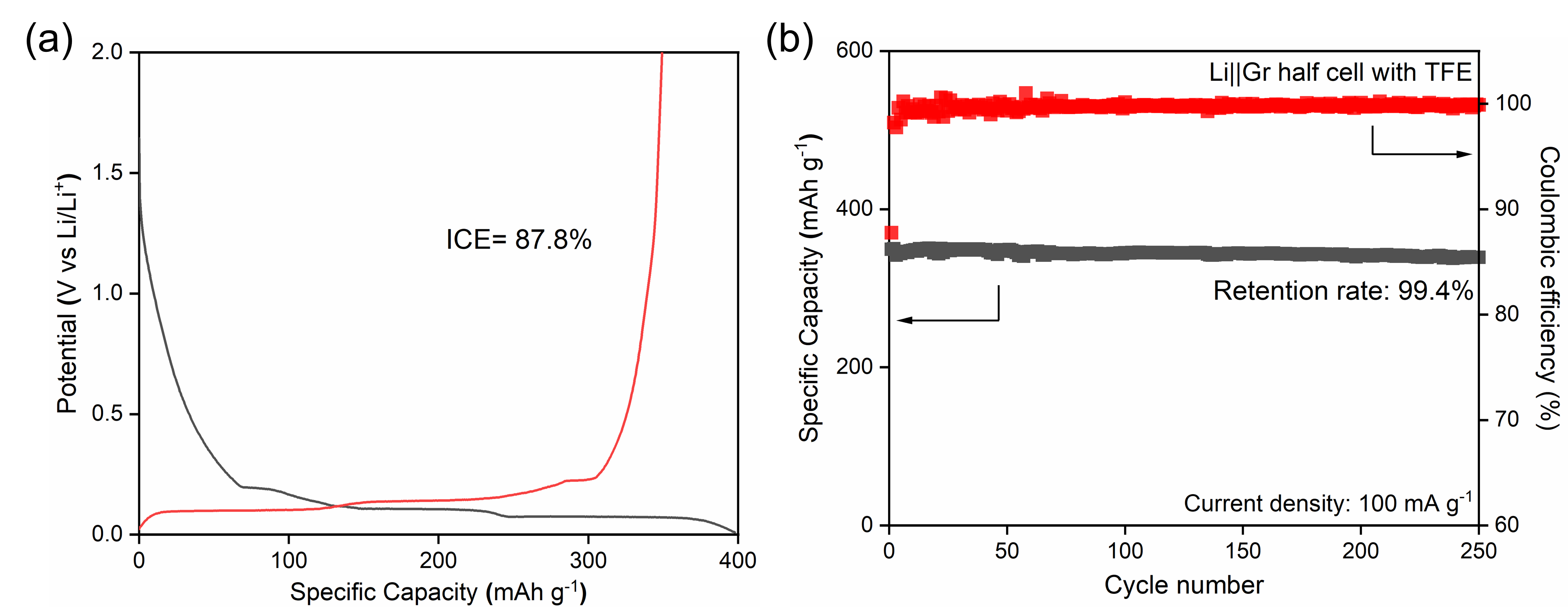


**Fig. S21** (**a**) First charge and discharge curve of Gr anodes in TFE electrolyte under a current density of 25 mAh g^-1^; (**b**) Cycling performance and Coulombic efficiency of Li/Gr half cells with TFE electrolyte. The specific current density is 25 mAh g^-1^ in the first three cycles and 100 mA g^−1^ in the subsequent cycles. The voltage range is 0.01–2.0 V (vs. Li/Li^+^).


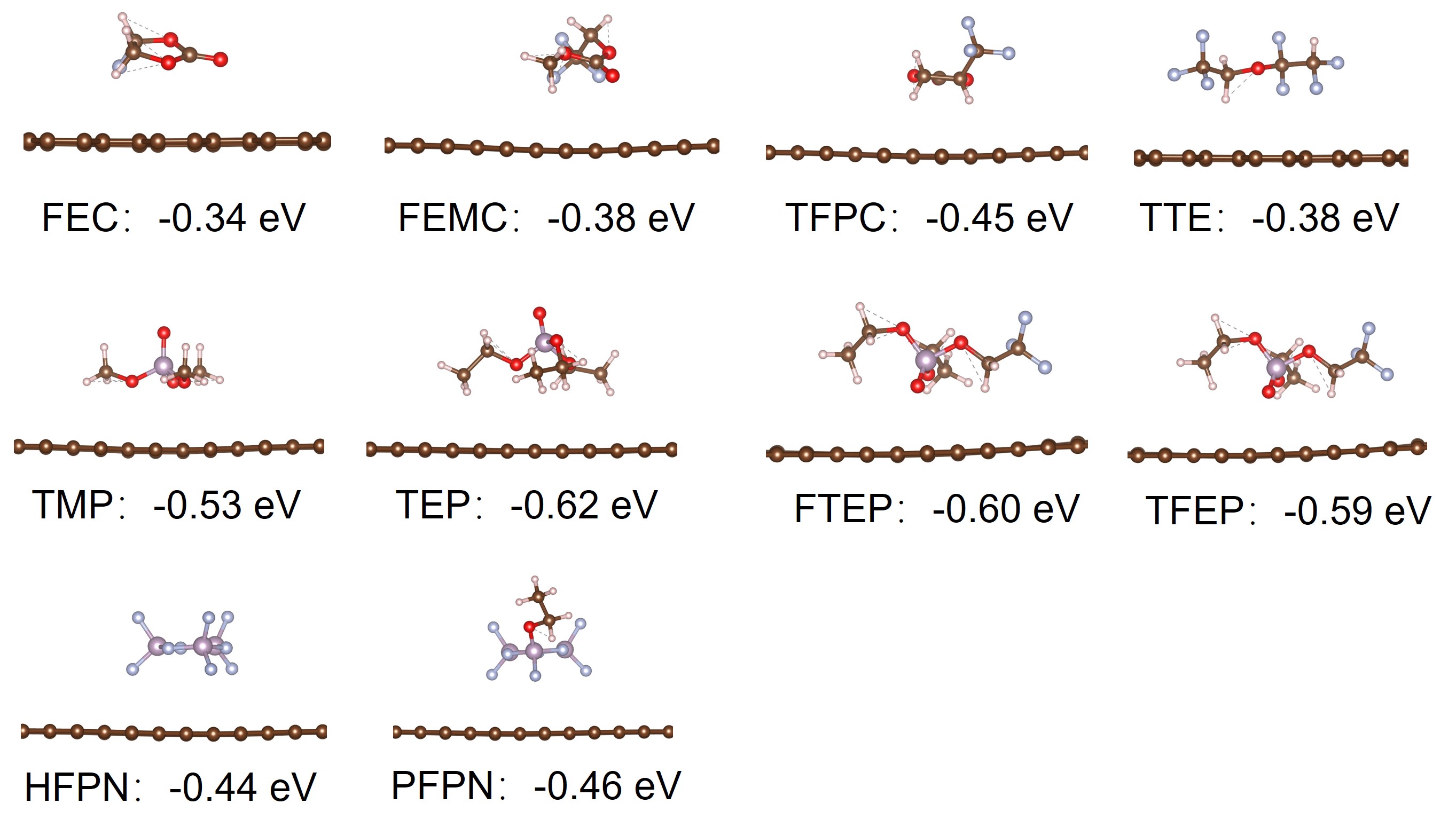


**Fig. S22** The absorption energies and models of ten typical flame-retardant solvents on graphite surface


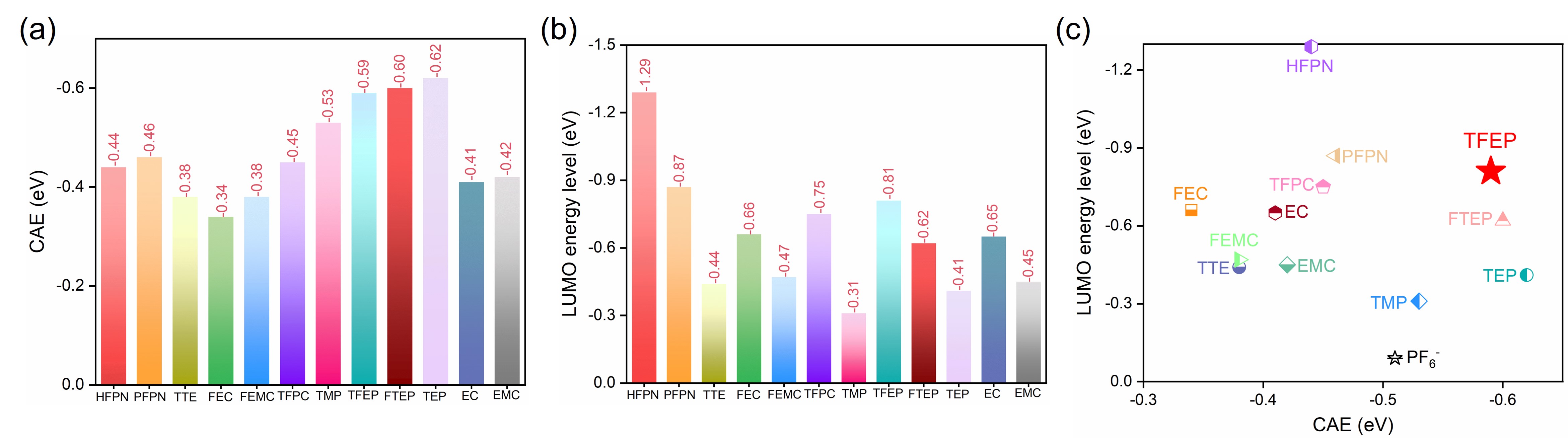


**Fig. S23** (**a-b**) CAE and LUMO energy level of different solvents; (**c**) Two-dimensional diagrams for solvent screening


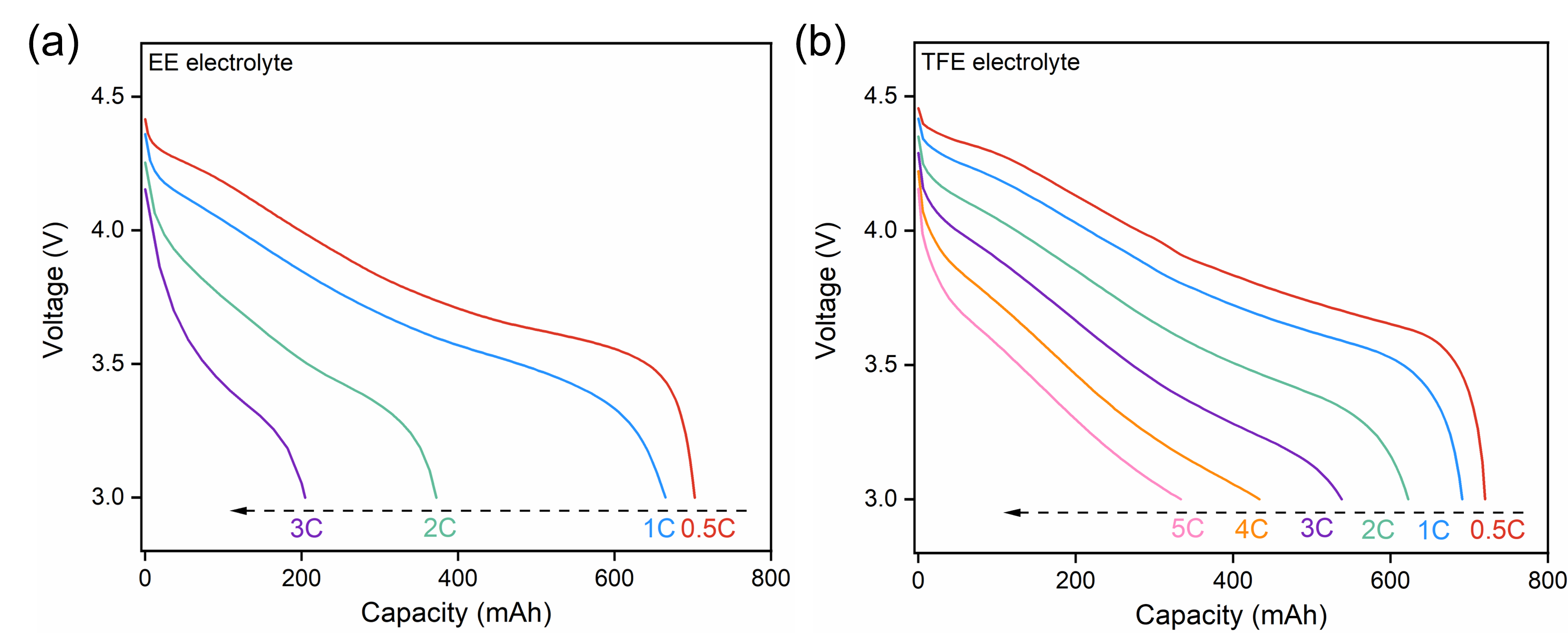


**Fig. S24** Discharge curves of 0.7 Ah Gr||LCO pouch cells with (**a**) EE and (**b**) TFE electrolyte at different current rates


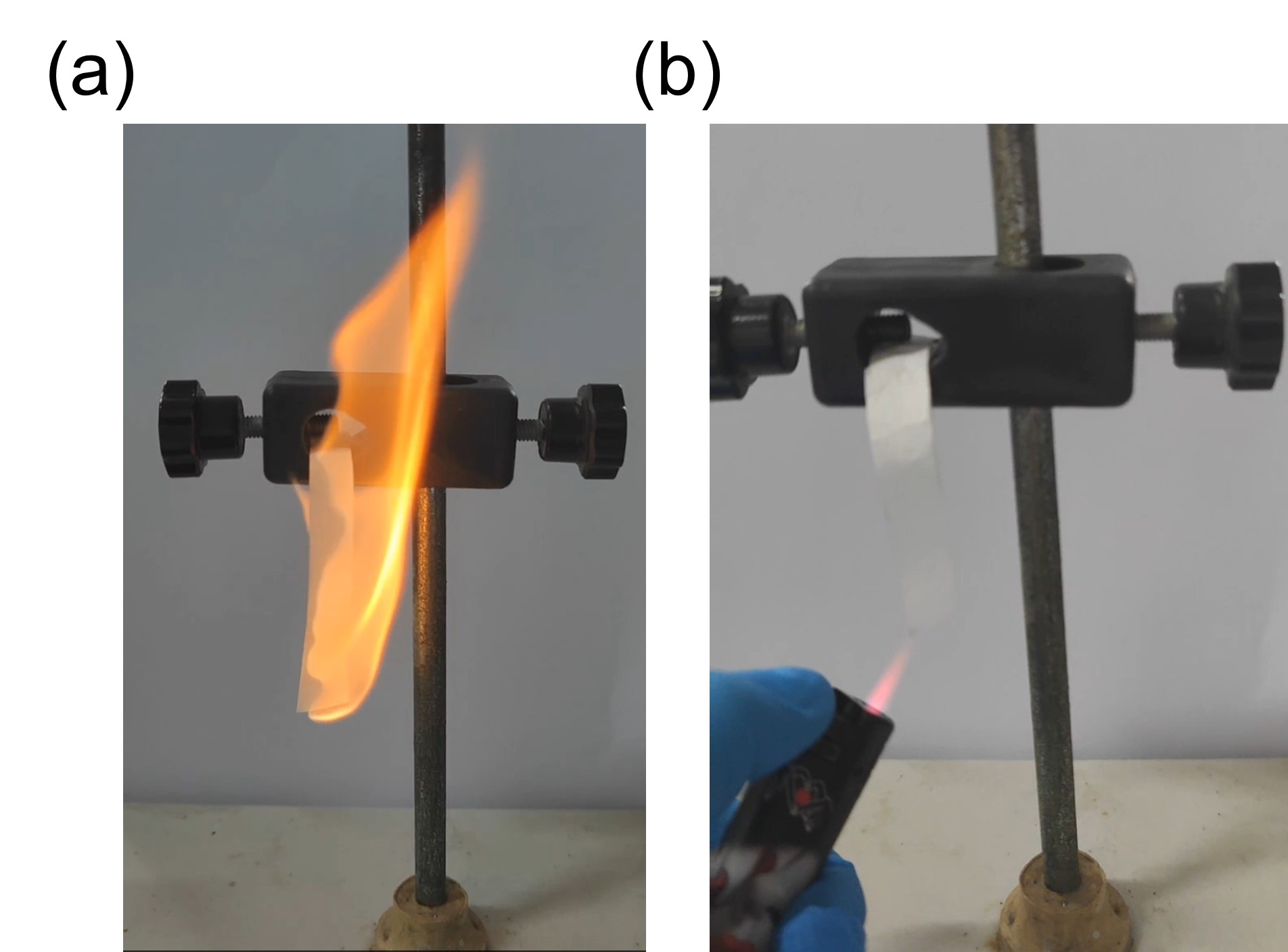


**Fig. S25** Flame tests of (**a**) EE and (**b**) TFE electrolytes


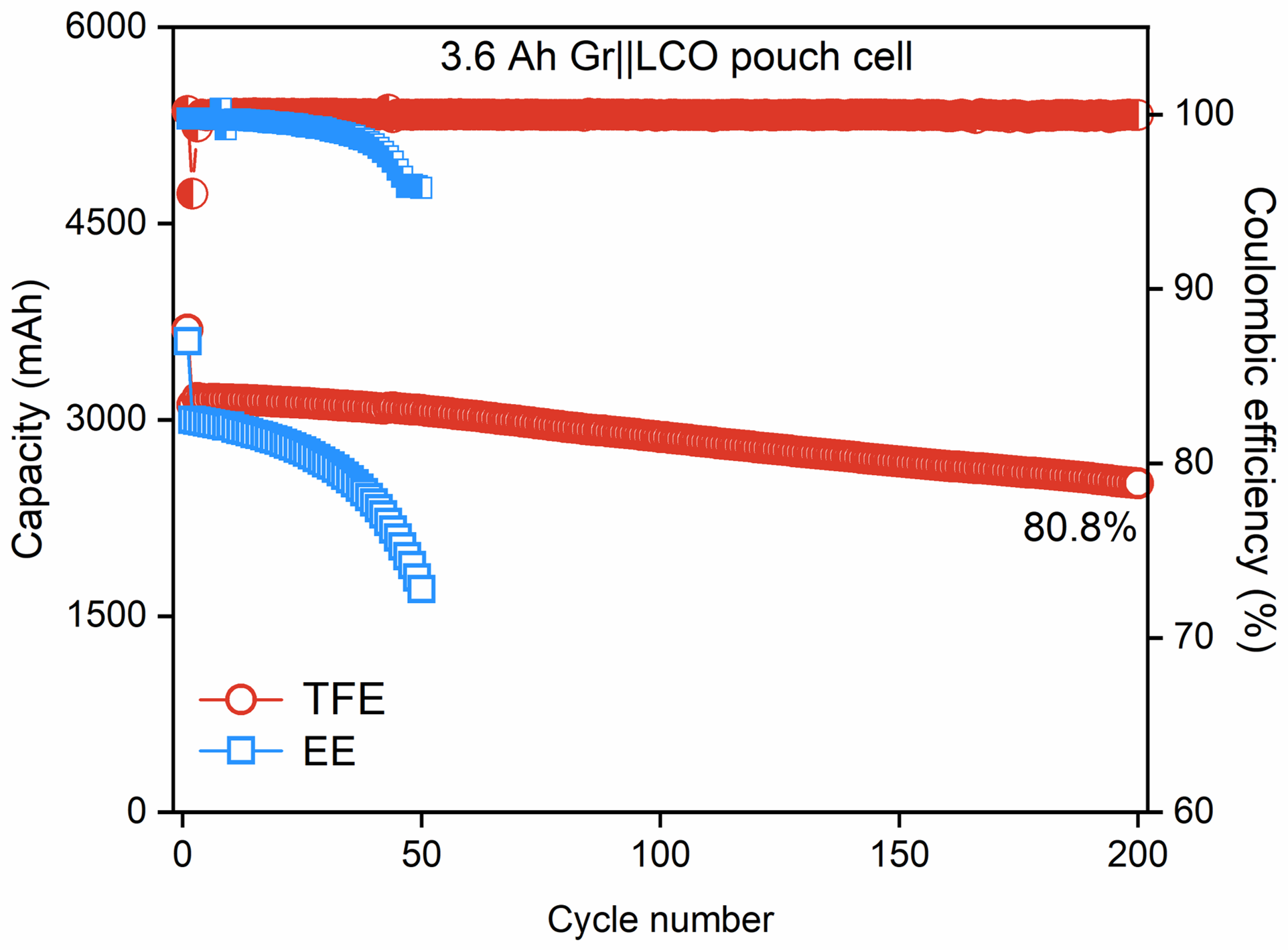


**Fig. S26** Cycling performance and Coulombic efficiencies of 3.6 Ah Gr||LCO pouch cells with EE and TFE electrolytes under a current of 0.5 C (1C= 3600 mA)


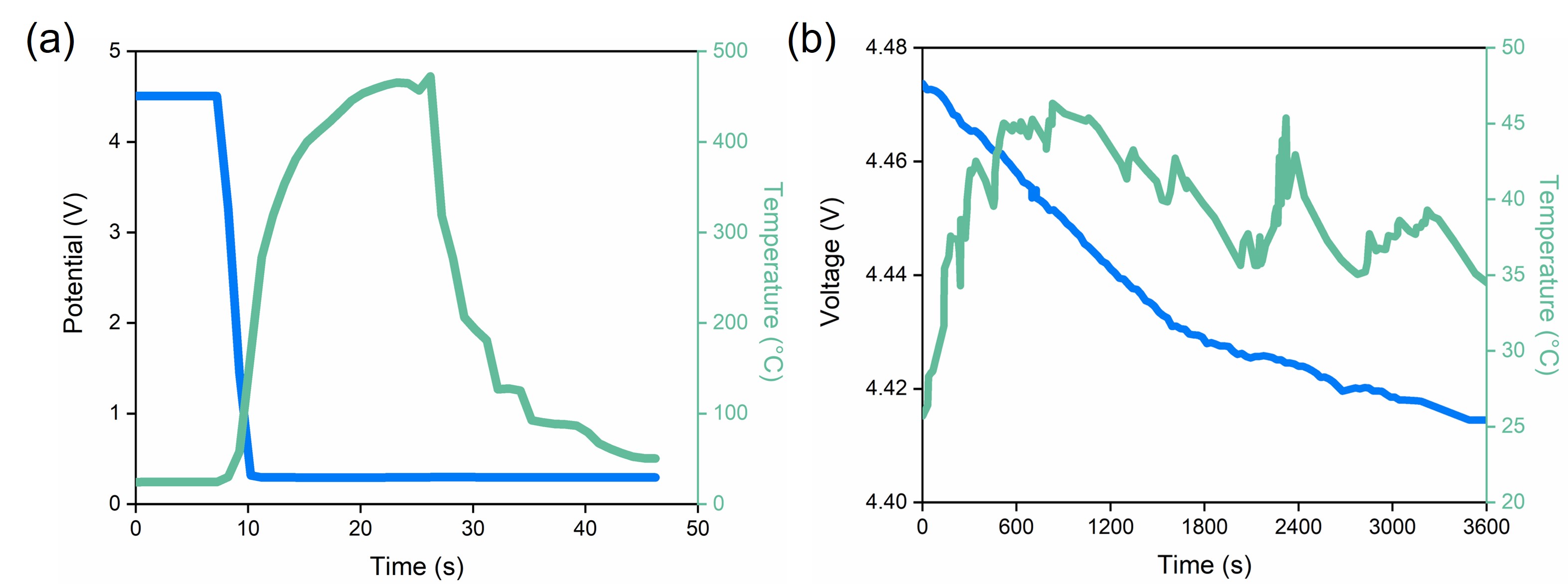


**Fig. S27** Time-voltage/temperature curves of Gr||LCO pouch cells during nail penetration using (**a**) EE electrolyte and (**b**) TFE electrolyte


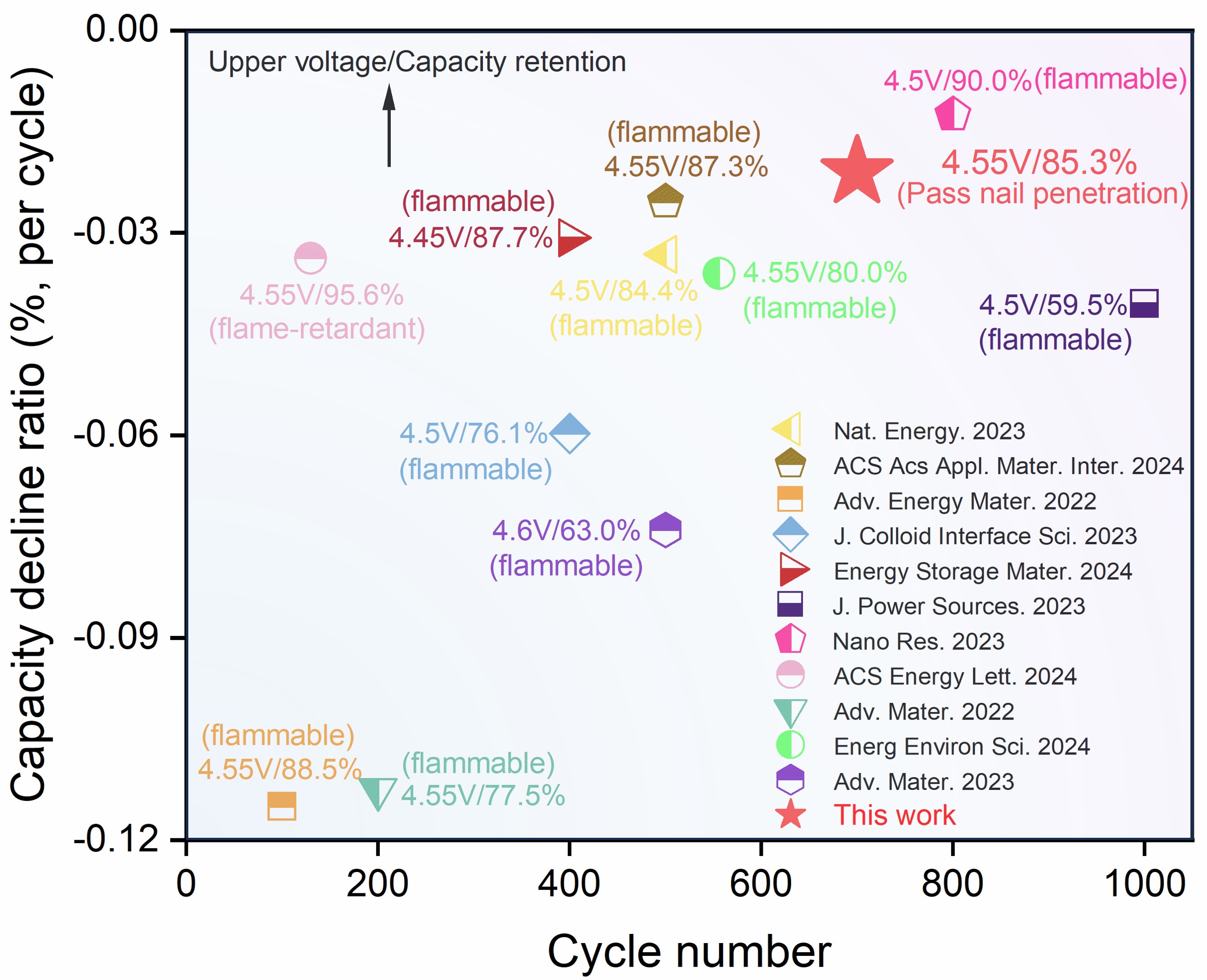


**Fig. S28** Comparison of electrochemical performance of recently reported high-voltage LCO cathodes


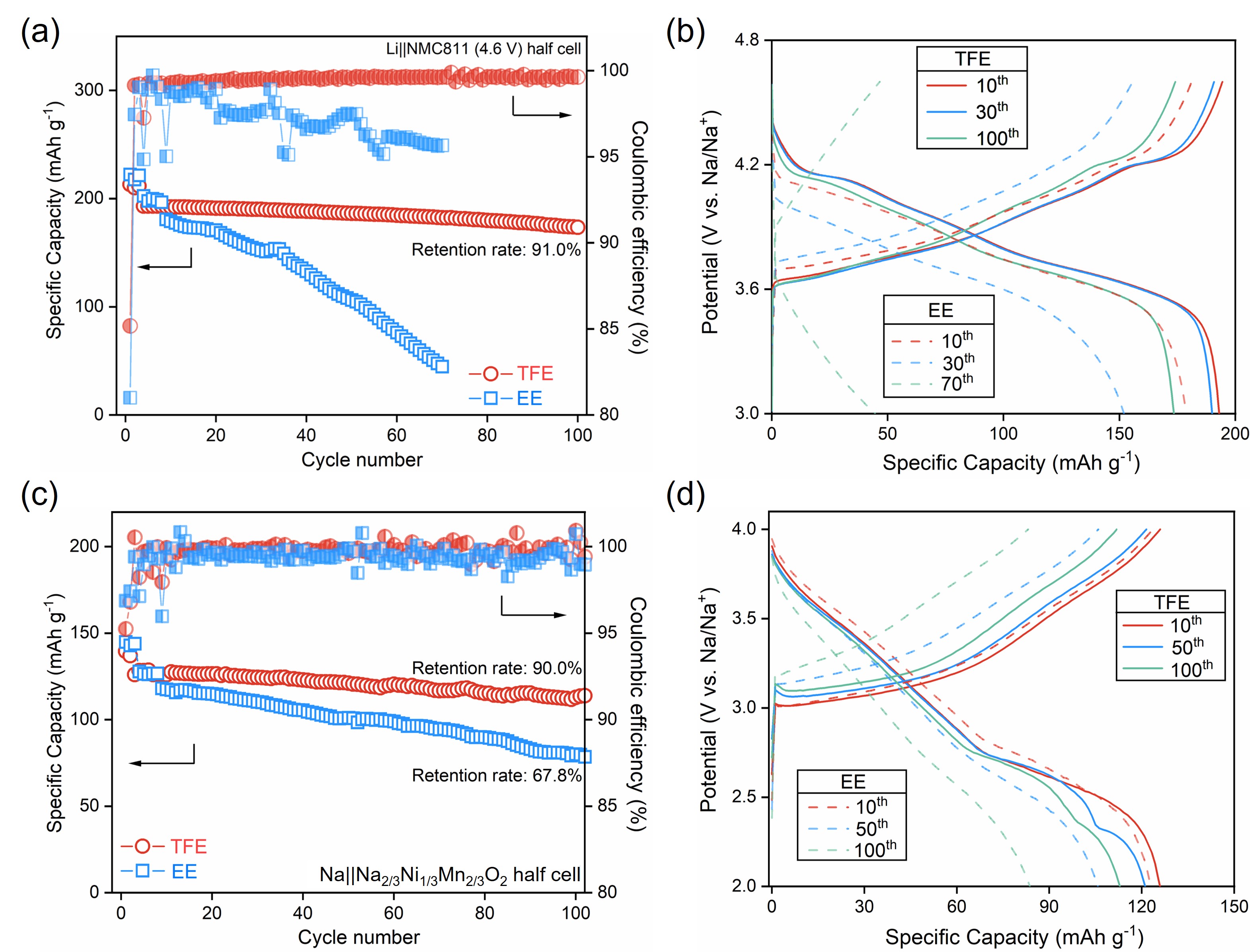


**Fig. S29** (**a-b**) Cycling performance and charge-discharge curves of Li||NMC811 half-cells with TFE and EE electrolyte in a voltage range of 3.0-4.6 V; (**c-d**) Cycling performance and charge-discharge curves of Na||Na_2/3_Ni_1/3_Mn_2/3_O_2_ half-cells with NaPF_6_-TFEP/EMC and NaPF_6_-EC/EMC in a voltage range of 2.0-4.0 V


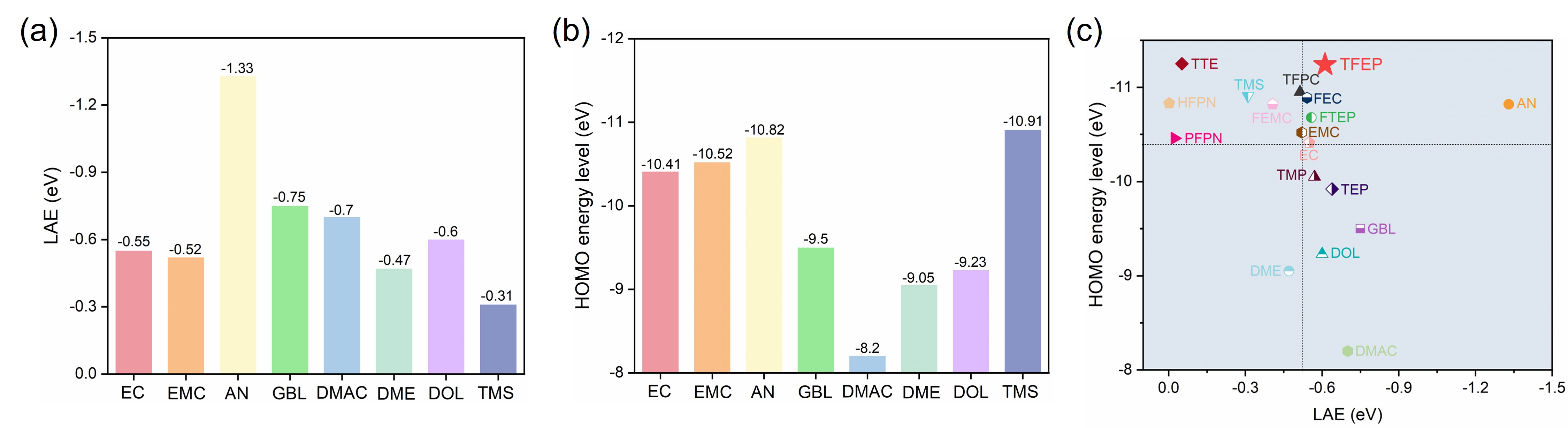


**Fig. S30** (**a-b**) LAE and HOMO of different solvents; (**c**) Two-dimensional diagrams for solvent screening, where *x* axis is LAE and *y* axis is HOMO

**Table S1** The physical properties of selected solvents

|  | **Viscosity (mPa·s)** | **Density (cm^3^ g^-1^)** | **Melting point (****℃)** | **Flash point (℃)** |
| --- | --- | --- | --- | --- |
| Hexafluorocyclotriphosphazene  (HFPN) | / | 2.24 | 25-30 | 60.9 |
| Ethoxy(pentafluoro)cyclotriphosphazene (PFPN) | / | 2.08 | -34 | 85 |
| 2,2,3,3-Tetrafluoro-1-(1,1,2,2-tetrafluoroethoxy)propane (TTE) | 0.25 | 1.53 | -94.3 | 27.5 |
| Methyl 2,2,2-trifluoroethyl carbonate (FEMC) | 0.54 | 1.31 | -44.2 | 65 |
| 3,3,3-Trifluoropropylene carbonate (TFPC) | 3.0 | 1.55 | / | >120 |
| Fluoroethylene carbonate (FEC) | 1.9 | 1.45 | 18 | >102 |
| Trimethyl phosphate (TMP) | 1.2 | 1.21 | -46 | 107 |
| Triethyl phosphate (TEP) | 1.6 | 1.07 | -56.5 | 117 |
| 2,2,2-Trifluoroethyl phosphate (FTEP) | / | / | / | >120 |
| Tris(2,2,2-trifluoroethyl) phosphate (TFEP) | 3.1 | 1.56 | -22 | >130 |
| Ethylene carbonate (EC) | 1.9 | 1.32 | 36.5 | 152 |
| Ethyl methyl carbonate (EMC) | 0.65 | 1.0 | -55 | 23 |
| Acetonitrile (AN) | 0.34 | 0.79 | -45 | 2 |
| Gamma-butyrolactone (GBL) | 1.5 | 1.13 | -43.5 | 98 |
| Dimethylacetamide (DMAC) | 0.92 | 0.94 | -20 | 70 |
| Dimethoxyethane (DME) | 0.32 | 0.87 | -58 | -2 |
| 1,3-Dioxacyclopentane (DOL) | 0.57 | 1.01 | -95 | 1 |
| Tetramethylene sulfone (TMS) | 10.29 | 1.26 | 27.5 | 166 |

**Table S2** The fitted EIS parameters of the LCO cathode at different cycles in EE and TFE electrolytes

|  | ***R*_CEI_ (Ω)** | ***R*_ct_ (Ω)** |
| --- | --- | --- |
| EE (pristine) | / | 164.9 |
| EE (1st cycle) | 203.5 | 224.3 |
| EE (50th cycle) | 527.8 | 418.4 |
| TFE (pristine) | / | 206.8 |
| TFE (1st cycle) | 49.8 | 61.7 |
| TFE (50th cycle) | 53.4 | 63.0 |
